# Shedding Light on the Chemistry and the Properties of Münchnone Functionalized Graphene

**DOI:** 10.3390/nano11071629

**Published:** 2021-06-22

**Authors:** Giulia Neri, Enza Fazio, Antonia Nostro, Placido Giuseppe Mineo, Angela Scala, Antonio Rescifina, Anna Piperno

**Affiliations:** 1 Department of Chemical, Biological, Pharmaceutical and Environmental Sciences, University of Messina, V.le F. Stagno d’Alcontres 31, 98166 Messina, Italy; giulia.neri@unime.it (G.N.); anostro@unime.it (A.N.); ascala@unime.it (A.S.); 2 Department of Mathematical and Computational Sciences, Physics Science and Earth Science, University of Messina, V.le F. Stagno d’Alcontres 31, I-98166 Messina, Italy; 3 Department of Chemical Sciences, University of Catania, V.le A. Doria, 95125 Catania, Italy; gmineo@unict.it; 4 Department of Drug and Health Sciences, University of Catania, V.le A. Doria, 95125 Catania, Italy

**Keywords:** nisin, antibacterial graphene, silver nanoparticles, 1,3-dipolar cycloaddition, münchnone, oxazolone, graphene density functional theory (DFT) calculation, reduced graphene, mesoionic compounds

## Abstract

Münchnones are mesoionic oxazolium 5-oxides with azomethine ylide characteristics that provide pyrrole derivatives by a 1,3-dipolar cycloaddition (1,3-DC) reaction with acetylenic dipolarophiles. Their reactivity was widely exploited for the synthesis of small molecules, but it was not yet investigated for the functionalization of graphene-based materials. Herein, we report our results on the preparation of münchnone functionalized graphene via cycloaddition reactions, followed by the spontaneous loss of carbon dioxide and its further chemical modification to silver/nisin nanocomposites to confer biological properties. A direct functionalization of graphite flakes into few-layers graphene decorated with pyrrole rings on the layer edge was achieved. The success of functionalization was confirmed by micro-Raman and X-ray photoelectron spectroscopies, scanning transmission electron microscopy, and thermogravimetric analysis. The 1,3-DC reactions of münchnone dipole with graphene have been investigated using density functional theory to model graphene. Finally, we explored the reactivity and the processability of münchnone functionalized graphene to produce enriched nano biomaterials endowed with antimicrobial properties.

## 1. Introduction

**Figure 1:**
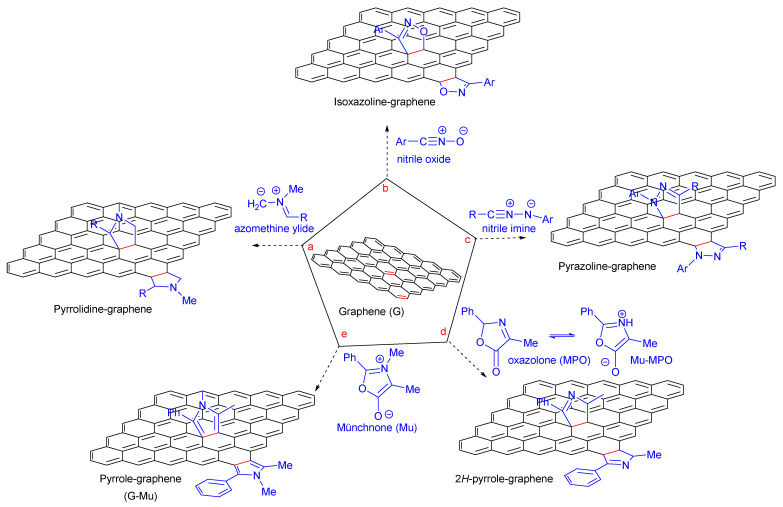
Representative examples of 1,3-DC reaction on graphene (G): (**a**) 1,3-DC of azomethine ylide to G [13]; (**b**) 1,3-DC of nitrile oxide to G [14]; (**c**) 1,3-DC of nitrile imine to G [15]; (**d**) 1,3-DC of oxazolone to G [5]; and (**e**) 1,3-DC of münchnone to G reported herein.

During the last decade, several groups have pioneered cycloaddition chemistry for the chemical modification of a wide array of carbon-based nanomaterials, including fullerene, carbon nanotubes, graphene, and carbon quantum dots, either in their pristine or functionalized forms [1,2,3,4,5]. The different cycloaddition strategies are an important tool to increase the processability, solubility, and colloid stability of nanostructures, to achieve better control over composition, and to target specific applications [6,7,8,9,10,11,12]. Among the different types of cycloaddition reactions employed for the covalent functionalization of the graphene sp^2^ network, it is arguable that the 1,3-dipolar cycloaddition (1,3-DC) and, in particular, the cycloaddition of azomethine ylides, is the most heavily used. The 1,3-DC of azomethine ylides on the graphene layer was exploited to introduce fused pyrrolidine rings on both the basal plane and the edge of the graphene sheet (Figure 1, pathway a). The dipole azomethine ylides were obtained by the condensation of an amino acid and an aldehyde under moderate–high temperature (above 80 °C) [13]. Although computational studies have predicted, in some cases, difficulties in the reaction progress on graphene for dipoles different from azomethine ylide, several successful examples of 1,3-DC reactions of different dipoles have been currently reported in the literature. The 1,2-heterobifunctionalized graphene was obtained by the reaction of nitrile oxides under microwave (MW) irradiation (Figure 1, pathway b) [14]. The formation of pyrazolines on graphene was achieved by 1,3-DC of nitrile imines (Figure 1, pathway c). The reaction proceeded by forming nitrile imine dipole intermediate obtained from the precursor hydrazones, in the presence of *N*-chlorosuccinimide [15].

The chemistry and the reactivity of mesoionic compounds have been extensively investigated by our research group in the last years [16,17,18,19], including the thermally induced addition of mesoionic (dipole) oxazolones to graphite flakes under solvent-free conditions for the direct production of a graphene sheet decorated with 2H-pyrrole derivatives (Figure 1, pathway d) [5].

Theoretical calculations indicated that the 1,3-DC reaction might proceed through a concerted mechanism or a competing stepwise mechanism, and the irreversible decarboxylation in the last stage represents the driving force of the process [5]. Moreover, our studies suggested that the preformed münchnones (Mu), skipping the tautomerization process, could be more reactive in 1,3-DC, affording the new pyrrole functionalized graphene. In this paper, we describe the preparation of pyrrole-decorated few-layer graphene (G-Mu) via 1,3-DC of mesoionic oxazolium 5-oxides (münchnone) in the presence of graphite flake dispersion (Figure 1, pathway e). The 1,3-DC strategies reported in the literature and described in Figure 1 break up the sp^2^ network of the graphene layer due to the not-planar structure of grafted heterocycles, whereas pyrrole grafting will produce an expansion of the graphene layer and the introduction of the nitrogen atom according to our proposed strategy (Figure 1, pathway e). Moreover, doping graphene with nitrogen could modify the material’s electronic and electrochemical properties, leading to enhanced performance [20].

Several physicochemical characterization techniques and surface analysis have been exploited to demonstrate successful functionalization. The mechanism involving 1,3-DC has been rationalized based on both theoretical and experimental results.

Finally, diazonium chemistry was exploited to graft benzene thiol groups on G-Mu surfaces; the resulting functionalized graphene was combined with the antimicrobial peptide Nisin interfaced at silver nanoparticles (Ag NPs) to obtain graphene enriched antimicrobial nano biomaterials. Aryl diazonium salts are privileged modifiers of materials surfaces due to their ease of preparation, the wide choice of reactive functional groups, the compatibility with a wide range of materials, and the strong aryl–surface covalent bonding [21,22]. The grafting of aryl radicals on carbon-based materials prevents the aggregation phenomena and improves their dispersibility. Moreover, the functional group in the para position of the aryldiazonium ring can affect the processability enabling the interaction with polymers, proteins, metal nanoparticles, etc., by noncovalent interactions or covalent bonds [23,24]. Nisin was selected as a component of the antimicrobial nanocomposite since it is a safety lantibiotic (i.e., a group of peptidic antibiotic containing unusual amino acids such as lanthionine residues) approved by regulation agencies (i.e., DFDA, EMA) as a food preservative for its activity against Gram-positive microorganisms [25]. Nisin silver nanoparticle assembly (Nisin@Ag) was proposed as a potential antibacterial formulation to broaden the efficacy of nisin towards Gram-negative microorganisms. The antibacterial activity of the new nanocomposite was assayed against selected Gram-positive (*Staphylococcus aureus*) and Gram-negative (*Escherichia coli*, *Pseudomonas aeruginosa*) bacteria.

## 2. Materials and Methods

### 2.1. Materials

Graphite flakes (GF), *N*-benzoyl-*N*-methylalanine, acetic anhydride, 4-aminothiophenol, silver nitrate, commercial nisin (~2.5% *w*/*w*) and solvents were purchased from Merck (Milan, Italy) and used as received.

### 2.2. Characterization Techniques

X-ray photoelectron spectroscopy (XPS) spectra were collected using the Thermo Scientific K-Alpha system (Gloucester, UK), equipped with a monochromatic Al Kα source (1486.6 eV) and a hemispherical analyzer operating in constant-pass energy (CAE) mode. For survey scans and the XPS core-level spectra, the pass energy was set at 200 eV and 50 eV, respectively. The core-level spectra were subtracted by a Shirley-type background. Moreover, to calibrate the charging effect, the spectra were referenced to the peak position of C 1s core levels to 284.5 eV.

Raman spectra were acquired by the Horiba XploRA spectrometer (Montpellier Cedex, France) using an 1800 groves mm^−1^ grating and a Peltier cooled CCD sensor (1024 × 256 pixels). The samples were excited with the 638 nm line from a solid-state laser, and all the spectra were collected using a 50 s acquisition and 80 scans for signal averaging, and a 50× microscope objective.

The Zeiss-Gemini 2 scanning electron microscope (Hamburg, Germany), also operating in transmission mode (STEM) at 150 and 30 kV, was used to analyze the samples’ morphology, and determine the number of graphene layers and their orientation with respect to each other. The Sonics VCX 130 ultrasonic sonicator allows dispersing some drops of the samples in isopropanol (0.5 mg/mL). The samples so treated were deposited on a 400-mesh holey carbon-coated copper grid and left to dry at room temperature for 2 h before carrying out STEM measurements.

X-ray diffraction analysis (XRD) was used to investigate the structural characteristics of the samples using the Bruker AXS D8 Advance (x-ray wavelength of 1.5418 Angstrom, Karlsruhe, Germany).

Thermogravimetric analyses (TGA) were performed by using Perkin–Elmer Pyris TGA7 (temperature range of 50–800 °C, Waltham, MA, USA). About 5 mg of each sample was placed in a platinum pan until balance stabilization and then heated with a scan rate of 10 °C/min under N_2_ flux. The balance sensitivity was 0.1 mg. A baseline recorded in the same measurement conditions with an empty platinum pan was subtracted from each thermogram before data analysis.

### 2.3. Synthesis of Münchnone Functionalized Graphene

GF (100 mg) was homogeneously dispersed in toluene (20 mL) or dimethylformamide (DMF) by sonication. *N*-benzoyl-*N*-methylalanine (620 mg, 6 mmol) and acetic anhydride (507 μL, 6 mmol) were added, and the reaction mixture was left to stir for 4 h at 60 °C. Afterward, it was cooled to r.t. and diluted with MQ water. Functionalized graphene was recovered by filtration under vacuum (Millipore 0.1 µm). It was washed three times with ethanol, and each time was sonicated for 10 min and separated from the supernatant by centrifugation at 3500× *g* rpm for 20 min. The effective elimination of unreacted dipole was monitored by NMR analysis of washing solvents. Finally, the residue was dried at ~60 °C to obtain 82 mg of functionalized graphene. The samples obtained using toluene or DMF as a solvent for 1,3-DC were, namely, G-Mu1 and G-Mu2, respectively.

### 2.4. Synthesis of Thiol Münchnone Functionalized Graphene

Sodium nitrite (86 mg, 1.22 mmol) was slowly added to a solution of 4-aminothiophenol (140 mg, 1.11 mmol) in HCl 37% (21.5 mL), at 0 °C under stirring. Then, HCl 20% (556 μL) was added dropwise, and the reaction was stirred at 0 °C for 1 h to obtain the diazonium salt. At the same time, a homogeneous black suspension of G-Mu1 (60 mg) or G-Mu2 was prepared by sonication in MQ water (20 mL) at room temperature for 1 h. The diazonium salt solution was added to the black suspension, and the reaction mixture was sonicated at 0–5 °C for 6 h. The reaction mixture was diluted with MQ water and centrifuged at 4000× *g* rpm for 20 min. The supernatant was discharged, and the precipitate was collected and washed three times with a mixture of MQ water/methanol (1:1). Each purification step included sonication and centrifugation (4000× *g* rpm for 20 min). The residue was dried at 60 °C to give 55 mg of thiol münchnone functionalized graphene. The samples were namely G-Mu1SH and G-Mu2SH. The amount of phenyl-thiol moiety grafted on the graphene surface was estimated to be 3.1–4.0% ~ wt % by TGA analysis under a nitrogen atmosphere.

### 2.5. Synthesis of Nisin-Coated Ag NPs and Graphene Nisin-Coated Ag NP Nanocomposite

1 mL of AgNO_3_ solution (1 mg/mL) was added to a solution of commercially available nisin (10 mg, corresponding to 0.25 mg of peptide) in 10 mL of MQ water (10 mL). Then 0.2 mL of NaBH_4_ solution (5 mg/mL) was added, and the mixture was left under magnetic stirring for 2 h. The color of the solution turned reddish black.

G-MuSH/Nisin@Ag nanocomposite was prepared using a homogenous dispersion of G-Mu2SH (3.5 mg) in MQ water (10 mL) obtained by sonication using a microtip probe sonicator (60% power) under an ice bath (UW 2070 SONOPLUS, Bandelin Electronic, (Berlin, Germany) for 10 min. Commercially available nisin (10 mg, corresponding to 0.25 mg of peptide) and 1 mL of AgNO_3_ solution (1 mg/mL) were added under stirring. Then 0.2 mL of NaBH_4_ solution (5 mg/mL) was added, and the mixture was left under magnetic stirring for 2 h. The color of the solution turned to reddish black. Nisin@Ag and G-MuSH/Nisin@Ag were analyzed by UV-Vis optical spectroscopy. Silver and nisin concentrations of freshly prepared Nisin@Ag and G-MuSH/Nisin@Ag colloidal dispersions were estimated to be 90 and 22.3 µg/mL, respectively.

### 2.6. Antimicrobial Activity

The antimicrobial activity of Nisin@Ag and G-MuSH/Nisin@Ag was determined against representative Gram-positive and Gram-negative bacteria such as *Staphylococcus aureus* ATCC 6538, *Escherichia coli* ATCC 10536, and *Pseudomonas aeruginosa* ATCC 9027. The minimal inhibitory concentration (MIC) was evaluated by the broth microdilution method in round-bottomed microplates (96 wells), with reference to the guidelines of the Clinical and Laboratory Standards Institute (CLSI) [26]. Briefly, the samples were serially twofold-diluted in Mueller–Hinton broth (Oxoid, Mila, Italy) and inoculated with a final bacterial load of 5 × 10^5^ CFU/mL. The MIC was considered the lowest concentration that inhibited visible growth after incubation at 37 °C for 18 to 24 h. The minimal bactericidal concentration (MBC) was determined by subculturing from the non-turbid wells on Mueller–Hinton agar and was defined as the lowest concentration of sample that gave complete inhibition of colony formation on plates. A stock solution of commercially available nisin (18 mg/mL corresponding to 450 µg/mL of peptide) and AgNO_3_ were used as controls. The data from at least three replicates were evaluated. The samples were sterilized under a UV lamp for 2 h before incubation.

### 2.7. Computational Details

Geometry optimizations of the critical points (reactants, transition structures, and products) were studied at the (U)M06-2X level of theory [27,28]. For each geometry studied, calculation of harmonic vibrational frequencies was carried out at the same level of theory to characterize all structures as minima or transition states. For the minima, all the wavenumbers obtained are positive, whereas transition state structures were found to have only one negative eigenvalue, with the corresponding eigenvector involving the formation of the newly created bonds. Vibrational frequencies were calculated (1 atm, 298.15 K) for all optimized structures, at (U)M06-2X/6-31G(d) level of theory, and used, unscaled, to compute both ZPVE and thermal corrections at 298 K. The enthalpy and entropy changes were calculated from standard statistical thermodynamic formulas [29]. The intrinsic reaction coordinates [30,31] (IRC analysis) were also calculated to analyze the mechanism in detail for all the transition structures obtained at the same level of theory. All the optimizations were carried out by Berny’s analytic gradient method [32] included in the Gaussian 16 software package [33]. In all the cases, full geometry optimization was carried out without any symmetry constraints.

## 3. Results and Discussion

### 3.1. Synthesis and Characterization of Münchnone Functionalized Graphene

**Scheme 1:**
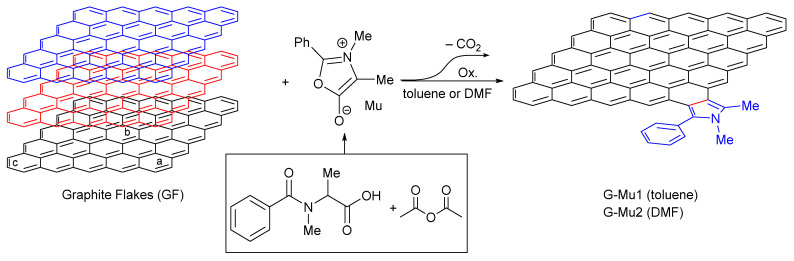
Schematic representation of the 1,3-DC reaction of münchnone with graphite flakes (GF).

**Figure 2:**
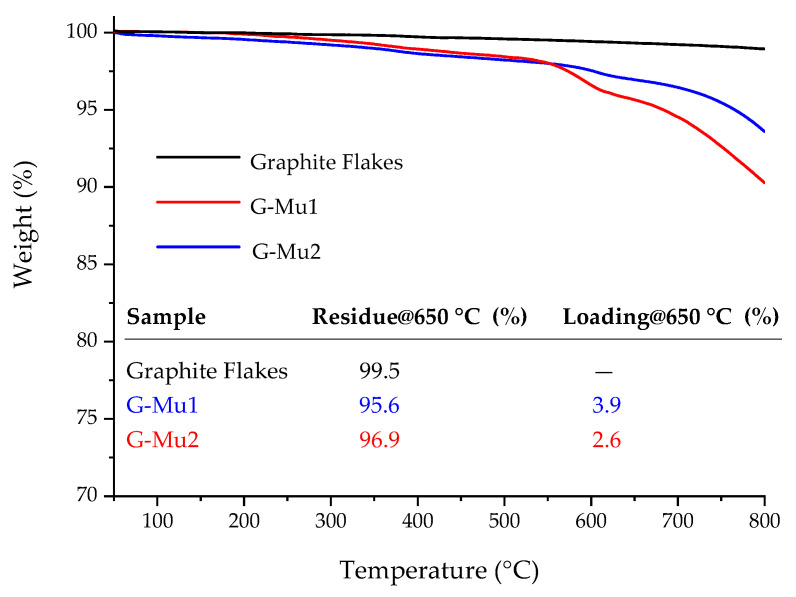
TGA profiles of graphite flakes (black line), G-Mu1 (red line), and G-Mu2 (blue line) in the N_2_ atmosphere. The inserted table reports the values, at 650 °C, of the weight residue (%) and loading (%).

**Figure 3:**
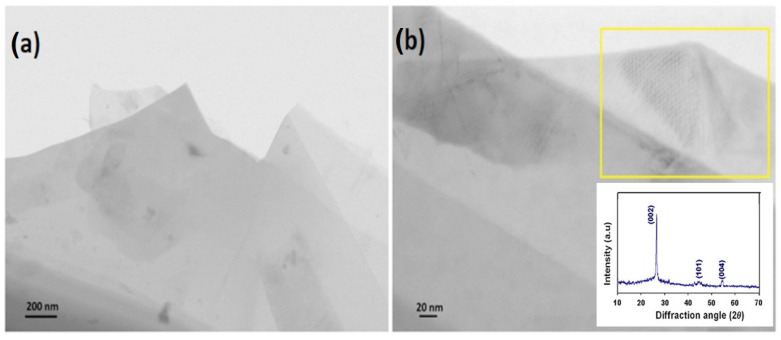
STEM image of a portion of G-Mu2 sample where various-dimensional transparent sheets stacked onto each other are evident (**a**); STEM image of G-Mu2 sample characterized by an highly crystalline structure (in particular see the yellow box and the inset reporting the G-Mu2 XRD spectrum) (**b**).

Münchnone (Mu) was generated in situ by a reaction between *N*-methyl benzoyl alanine and acetic anhydride. The cycloaddition reaction was performed in toluene or DMF at 60 °C by using the homogenously dispersed graphite flakes as dipolarophiles (Scheme 1). The functionalization degree (loading %) was estimated in terms of weight loss by TGA upon heating the solid reaction product in a nitrogen atmosphere (Figure 2). The reaction proceeded by simultaneous functionalization and delamination of graphite into few-layers graphene (as will be better discussed by analyzing the scanning transmission electron microscopy (STEM) images, see Figure 3).

The chemical composition and structural properties of functionalized graphene G-Mu1 and G-Mu2 were investigated by micro-Raman and X-ray photoelectron (XPS) spectroscopies, while the morphology was analyzed using the scanning transmission electron microscopy (STEM).

**Figure 4:**
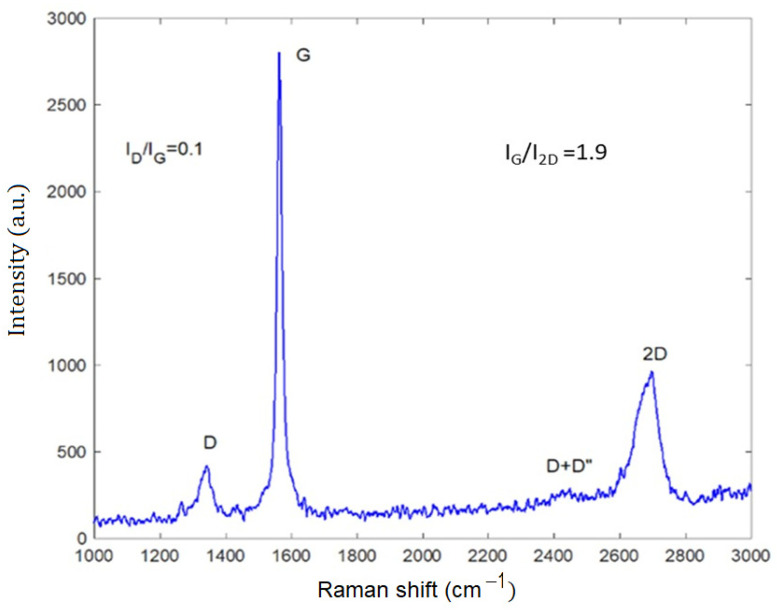
Raman spectrum of G-Mu2.

Representative STEM images and Raman spectrum of münchnone functionalized graphene are reported in Figure 3 and Figure 4, respectively. Specifically, STEM images of G-Mu2 (Figure 3a,b) show various-dimensional transparent sheets stacked onto each other, indicating the efficient functionalization and exfoliation of pristine graphite. In addition, the highly crystalline structure of the graphene-based material, obtained by the exfoliation of pristine graphite, is evident (see Figure 3b and XRD spectrum in the inset). The narrow intensive peak at 26.35° is highly specific for the crystalline nature of graphene, while the peaks at 44.35° and 54.23° are ascribed to the hexagonal structure typical of a graphite-like material (JCPDS # 411487).

The Raman spectrum is characterized by the well-known D band, at about 1350 cm^−1^, ascribed to the structural imperfections of graphene, the G band at about 1580 cm^−1^, followed by the G* band (commonly denoted as D + D″, resulting from the double-resonance Raman scattering processes) in the 2400–2450 cm^−1^ spectral region, and the second-order overtone 2D mode at about 2690 cm^−1^ [5] (Figure 4, the Raman spectrum of graphite is reported in S1). The I_D_/I_G_ and I_G_/I_2D_ peak intensities ratio are 0.1 and 1.9, respectively. The first ratio, which characterizes the level of disorder in the sample, is very low, indicating that the functionalization does not affect the pristine graphite structure. Further, the high I_G_/I_2D_ ratio and the wide 2D band indicate the presence of several graphene layers, in good agreement with the STEM analysis. Empirically, the G band position can be correlated to the number of atomic layers by the following relation: *w*_G_ = 1581.6 + 11/(1 + n^1.6^) where *w*_G_ is the band position in wavenumbers, and *n* is the number of sheets present in the sample. In our case, we estimated *n* = 3, in good agreement with STEM evidence. Furthermore, a thickness of about 2–3 nm was estimated.

**Figure 5:**
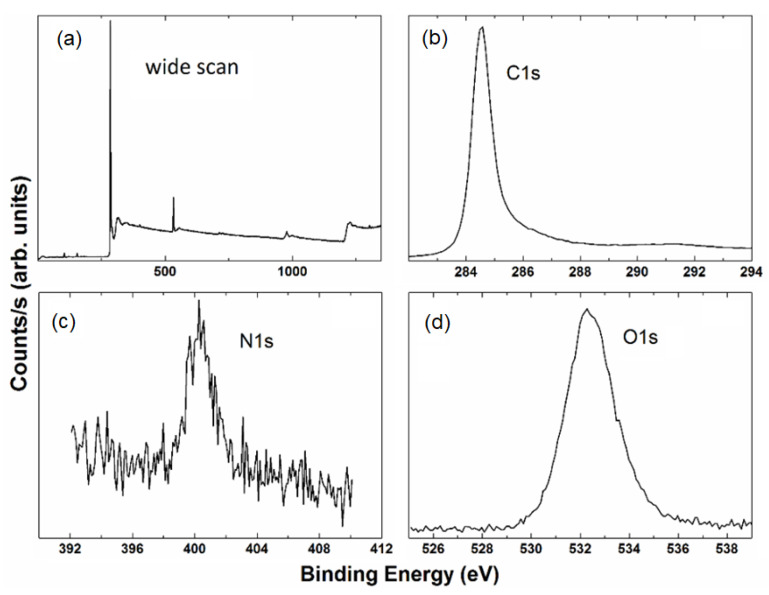
XPS spectra of G-Mu2. G-Mu2 wide scan (**a**) and the relative high-resolution C1s (**b**), N1s (**c**), and O1s (**d**) line shapes.

Information about the chemical composition of münchnone functionalized graphene was obtained by XPS analysis. Both G-Mu1 and G-Mu2 showed a similar chemical composition (Table 1), suggesting that the two different experimental conditions do not significantly influence the samples’ composition. The wide-scan XPS spectrum and the high-resolution spectra of C1s, O1s, and N1s core levels of G-Mu2 are reported in Figure 5. Carbon, oxygen, and nitrogen-related peaks are evident, together with a very low-intensity peak due to silicon from the sample substrate. The atomic percentage of the detected species was estimated by analyzing the high-resolution photoemission spectra, considering the relative species’ atomic Scofield’s sensitivity factor [34].

The C 1s spectrum is characterized by a broad band in the 283–290 eV region, typical of a carbon-based system. The main feature is a band peaked around at 284.5 eV and attributed to C=C/C–C in the aromatic ring, plus some other features resulting from the overlapping of higher binding energy bands. These contributions are ascribed to carbon atoms bonded to nitrogen (C–N), and oxygen in different surface functionalities (C–OH, C–O, C=O) centered at 285.2, 286.3, 288.7, 288.9, and 291 eV, respectively. The graphene functionalization is evidenced by the N1s profile, which is dominated by a broad band centered at about 400 eV due to nitrogen-carbon bonds. Finally, an O 1s broad band, characterized by the contribution from oxygen bond singly and doubly to carbon, centered at about 533.6 eV and 531.9, was also detected.

### 3.2. Computational Studies

According to our previous paper [5], we performed DFT calculation studies to rationalize the outcome of the 1,3-DC of Mu to GF. Briefly, one graphene layer was simulated by a polybenzenoid hydrocarbon consisting of 25 fused benzene rings (five in row and five in-line), terminated by hydrogen atoms, presenting zigzag-shaped edges (Scheme 1), that provides reliable energetics for both interior and peripheral bonds and represents a good compromise for computational investigations [35]. Although there are many types of double bonds in large graphene systems, we explored only three of them, defined as corner (a), center (b), and edge (c) (from here onward defined as pathways a–c, Scheme 1).

Due to the polyradical character of graphenes and their stability as open-shell singlet states [36,37,38], we performed unrestricted DFT calculations at the M06-2X level of theory employing the 6-31G(d) basis set, which performs very well with respect to the high demanding CCSD(T) method [39]. Although the two zigzag double bonds a and c are indistinguishable when the graphene model approaches infinite, in our simplified model, they are different. For the same reason, the double bonds b and c can lead to two regioisomeric products. Here we considered the formation of only one regioisomer, choosing, especially for c, the one with less steric hindrance [40]; at the same time, we have considered only the *exo* approach because refined computational results demonstrated that it is the preferred one [41], also in accord with our previous findings [5]. Moreover, we have not considered graphene defects because it was found that the presence of Stone–Wales translocations, 585 double vacancies, and 555–777 reconstructed double vacancies did not significantly change graphene reactivity [42]. Finally, considering that nitrogen inversion is a fast event, we have taken it into account and reported only the most stable conformer results.

**Figure 6:**
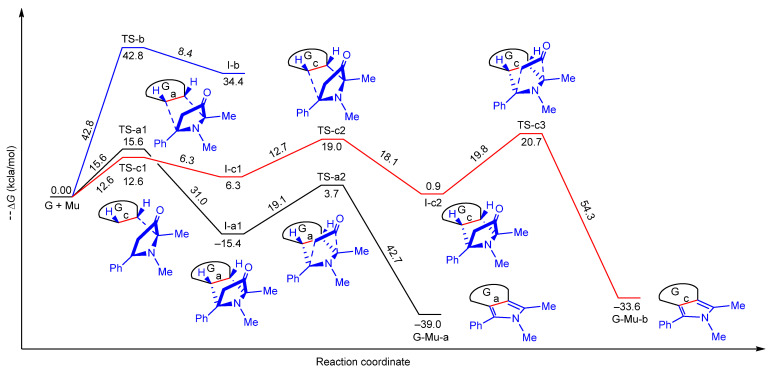
Free energy profiles for the a–c pathways involved in the 1,3-DC of 5 × 5 G model with Mu. All values refer to G + Mu.

The most important transition states (TS-a–c), intermediates (I-a–c), and product structures (G-Mu-a,c) have been schematized in Figure 6, whereas their energetic parameters have been summarized in Table 2 and Table S1.

The obtained results parallelize the ones obtained by us with the Mu-MPO [5]. For pathways a and b, we were able to localize only the concerted process, whereas, for pathway c, only the stepwise one was successful. Although alternative mechanisms might also be envisaged for both cases, all attempts to locate stationary points for reaction channels other than those herein reported were unsuccessful.

The inspection of Table 2 and Figure 6 highlights that pathway b is highly endergonic, according to the aromaticity disruption in four benzene rings. The formation of I-b can be ruled out. Pathway b, except for the decarboxylation process, is highly reversible, with the inverse Δ*G* barriers (6.3 and 18.1 kcal/mol for TSc1 and TS-c2, respectively) lower than the forward direct ones (12.7 and 19.8 kcal/mol). On the contrary, pathway a only proceeds forward. The concerted and irreversible decarboxylation stage, which is the slow step of the reaction, has a free activation energy of 0.7 kcal/mol, lower to the corresponding one in path c; thus, pathway a is effectively the only viable one.

### 3.3. Synthesis of Graphene Nisin-Coated Ag NPs Nanocomposite

**Scheme 2:**
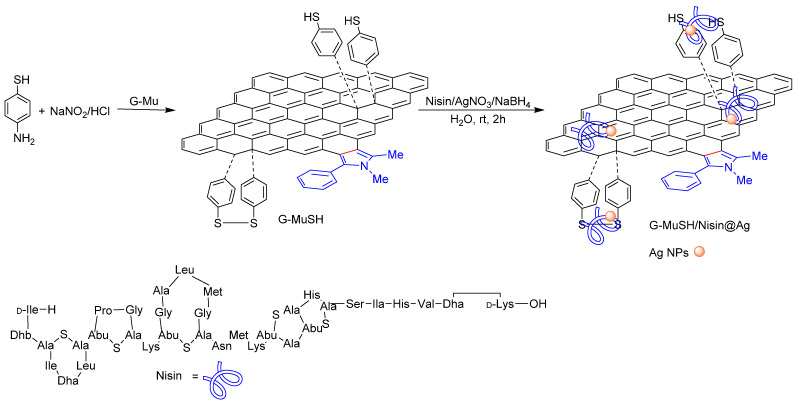
Schematic representation of G-MuSH/Nisin@Ag nanocomposite preparation.

The reactivity and the processability of münchnone functionalized graphene were exploited to produce enriched nano biomaterials with antimicrobial activity. Specifically, G-Mu was functionalized with benzene thiol groups by a reaction with the diazonium salts [43,44] prepared from 4-aminothiophenol, and used to produce the novel antibacterial graphene-based nanocomposite G-MuSH/Nisin@Ag (Scheme 2). We envisioned that benzene thiol groups grafted on G surfaces could provide anchorage to inorganic and organic antimicrobial agents such as Ag NPs and antimicrobial peptides. Among the family of antimicrobial peptides, we selected nisin as a model for our studies since it possesses a unique chemical structure characterized by three unusual amino acids (dehydroalanice, lanthionine, and *β*-methyl-lanthionine) and five internal sulfide bridges that, together with basic residues of Lys and Asp, contributed to stabilizing Ag NPs. Moreover, nisin is a heat-stable pentacyclic peptide, commercially available at low cost in its unpurified form [25,45].

**Figure 7:**
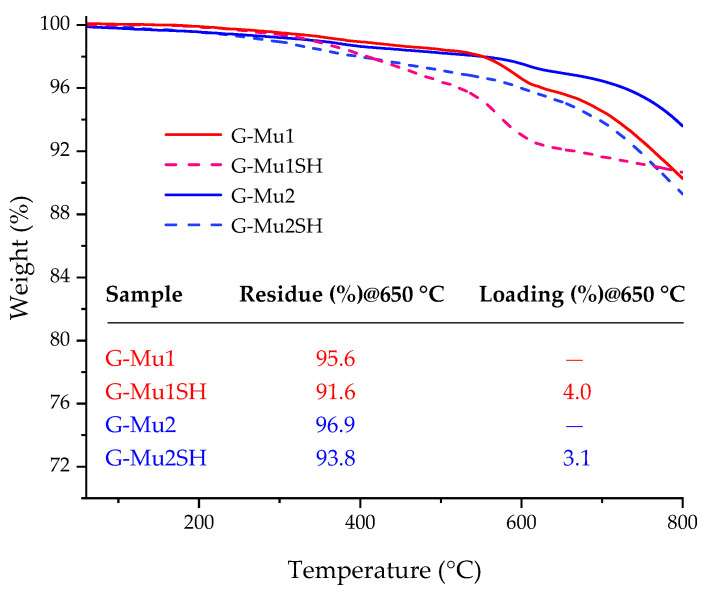
TGA profiles of G-Mu1 (red line), G-Mu2 (blue line), G-Mu1SH (red dashed line), and G-Mu2SH (blue dashed line) in a N_2_ atmosphere. The inserted table reports the values, at 650 °C, of the weight residue (%) and loading (%).

The success of benzene thiol groups grafting on G surfaces was proved by TGA (Figure 7). The TGA profiles of G-Mu1 and G-Mu2 show a constant mass loss up to 800 °C; whereas, G-MuSH profiles show an evident weight loss at 650 °C, with loading at 650 °C of 3.1% and 4.0% for G-Mu2SH and G-Mu1SH, respectively.

**Figure 8:**
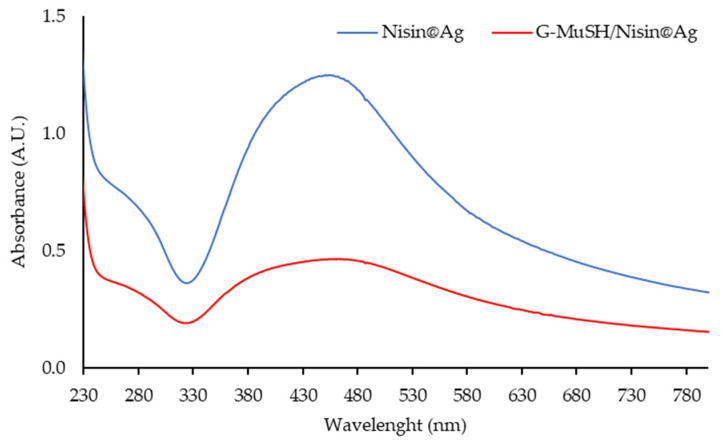
UV absorption spectra of freshly prepared Nisin@Ag [1.07 mg/mL] and G-MuSH/Nisin@Ag [1. 18 mg/mL].

According to the literature methods, NaBH_4_ was used in combination with a peptidic natural matrix to prepare Ag NPs [46,47]. Unlike other polymer matrices [46,48], nisin was found to be unable to reduce noble metal ions without NaBH_4_. Nisin acted as a stabilizing ligand to produce nisin-coated Ag NPs (Nisin@Ag), also in the presence of G-MuSH (G-MuSH/Nisin@Ag). In both the experiments, the color of the solution turned reddish-black after a few minutes, suggesting Ag nanostructure formation. The typical Ag plasmon resonance absorption feature, detected at about 430 nm for the Nisin@Ag sample, confirmed the Ag NPs formation (Figure 8). In addition, SPR lost most of its distinctive features in the G-MuSH/Nisin@Ag sample, due to the NPs size change and the effects of stabilizing molecules, which induce significant broadening of the extinction bands, as generally observed in the case of thiolated Ag NPs [49].

**Figure 9:**
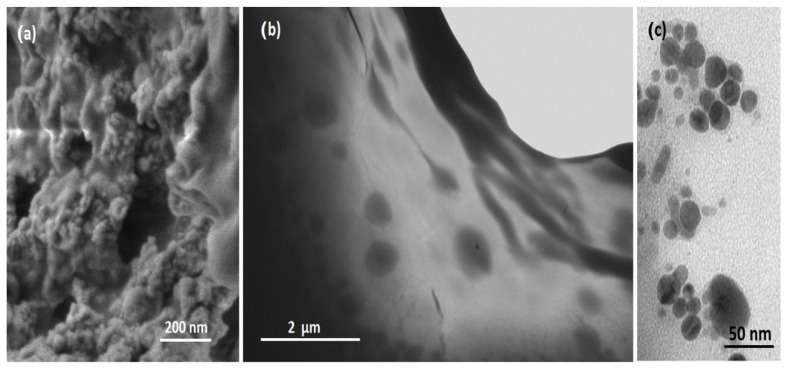
SEM (**a**) and STEM images (**b**,**c**) of the G-MuSH/Nisin@Ag sample.

Figure 9a shows that the MuSH/Nisin@Ag sample possesses the porous structure expected upon functionalizing the graphene surface with the nisin protein. Moreover, the presence of Ag NPs is evident from the STEM images shown in Figure 9b,c (a comparative STEM image of G-MuSH is reported in Figure S2). Ag NPs, with an average size of 20 nm, are evenly distributed on the G-MuSH surface, an indication of good interaction between graphene and Ag NPs through the nisin peptide.

**Figure 10:**
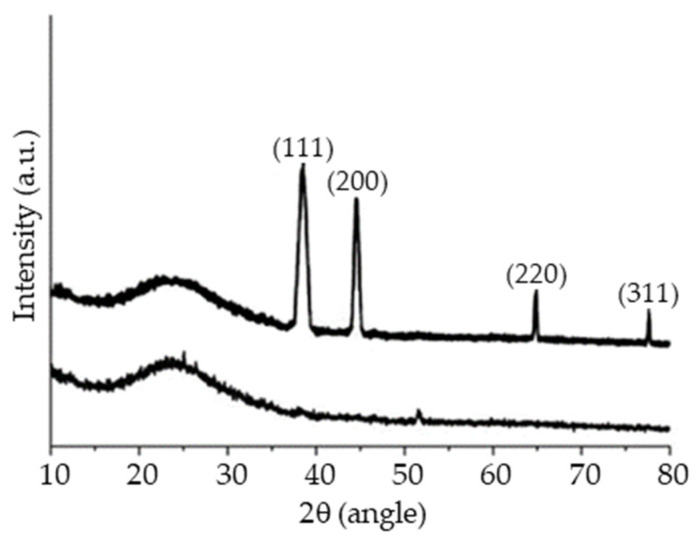
XRD spectra of G-MuSH (bottom) and G-MuSH/Nisin@Ag (top) samples.

XRD (Figure 10) is used for the determination of the crystal structure and lattice parameters. A broad diffraction peak at about 25.5° can be seen in the G-MuSH sample, a typical feature of reduced graphene oxide [50]. In addition, the XRD pattern of the G-MuSH/Nisin@Ag composite shows the characteristic peaks at about 38.2°, 44.5°, 64.6°, and 77.6°, assigned to the (111), (200), (220), and (311) crystallographic planes of the Ag face-centered cubic structure (JCPDS, file No. 04-0783), confirming the formation of Ag NPs in the composite.

### 3.4. Antimicrobial Activity

In recent years, the range of applications of Ag NPs, due to their irreplaceable properties (i.e., their small size and surface plasmonic properties), has been expanded from antibacterial drugs (proposed as a formulation with well-known antimicrobial agents) [47] to materials endowed with intrinsic antimicrobial properties [51,52], as well as for preparing light-responsive NP/nanocapsules employed as drug nanocarriers [53].

The antimicrobial activity of Nisin@Ag and G-MuSH/Nisin@Ag was analyzed against selected Gram-positive (*S. aureus*) and Gram-negative (*E. coli* and *P. aeruginosa*) bacteria (Table 3) using commercial nisin (containing ~2.5% *w*/*w* of active peptide) and AgNO_3_ as controls. MIC and MBC (minimal bactericidal concentration) values were evaluated (Table 3) to compare the bacteriostatic and bactericidal actions of Nisin@Ag and G-MuSH/Nisin@Ag.

Nisin showed antibacterial activity only against Gram-positive *S. aureus* with a MIC of 281.25 μg/mL, expressed as raw material, corresponding to 7.03 μg/mL of pure peptide. Interestingly, the contribution of silver in the Nisin@Ag interfacial assembly produced a broadening of the antibacterial spectrum to *E. coli* and *P. aeruginosa*, compared to nisin alone (Table 3). Since pure Ag NPs are unstable in aqueous solutions, and different factors can influence their antibacterial activity (i.e., capping agent, size, charge, plasmon band, etc.) [46,51], the contribution of silver ions was evaluated using AgNO_3_ as control. The effective antimicrobial action of AgNO_3_ was found in the range from 11.25 μg/mL to 2.8 μg/mL, and according to literature, silver ions in the form of AgNO_3_ are more potent antimicrobial agents than the Ag NPs [54].

The combination of Nisin@Ag with functionalized graphene (G-MuSH) afforded a nanocomposite endowed with antimicrobial activity but with doubled MIC values with respect to starting Nisin@Ag. The same behavior was observed for bactericidal activity. Due to the low dispersibility in water/dimethyl sulfoxide of pure münchnone functionalized graphenes (i.e., G-Mu and G-MuSH), the experiments to evaluate their antimicrobial activity failed. The formulation of G-MuSH with Nisin@Ag resulted in a doubly beneficial effect: the increase of münchnone functionalized graphene processability and the achievement of graphene enriched antimicrobial nano biomaterials.

## 4. Conclusions

In this work, we applied the chemistry of 1–3 dipolar cycloaddition to prepare few-layers graphene functionalized with pyrrole rings on the layer edges. The münchnone functionalized graphene was characterized by micro-Raman and X-ray photoelectron spectroscopies, scanning transmission electron microscopy, and thermogravimetric analysis. As inferred from theoretical calculations, the investigated 1,3-DC reaction proceeds through a concerted mechanism (pathway a) competing with a stepwise one (pathway b) involving two transition states plus a zwitterionic intermediate. However, the last stage is irreversible, and this behavior is in accord with the high degree of functionalization experimentally observed and represents the training force of the process. Benzene thiol groups were grafted on münchnone-graphene surfaces by diazonium chemistry and used to anchor inorganic and organic antimicrobial agents such as Ag NPs and nisin. The formulation of münchnone functionalized graphene with Nisin@Ag, as well as conferring antimicrobial properties to graphene material, produced increased processability. The G-MuSH/Nisin@Ag nanocomposite is a promising new nanomaterial with antimicrobial activity against both Gram-positive and Gram-negative bacteria.

## Figures and Tables

**Table 1:** Surface atomic composition expressed as percentages and obtained by XPS data.

	G-Mu1	G-Mu2	GF
C 1s (%)	93.1	92.7	88.4
O 1s (%)	5.6	6.4	11.6
N 1s (%)	1.3	0.9	0.0

**Table 2:** (U)M06-2X direct and inverse relative free energies (Δ*G*) (kcal/mol).

Structure	Direct Δ*G* ^a^	Inverse Δ*G* ^b^
TS-a1	15.6	31.0
TS-a2	19.1	42.7
TS-b	42.8	8.4
TS-c1	12.6	6.3
TS-c2	12.7	18.1
TS-c3	19.8	54.3
I-a1	−15.4	—
I-b	34.4	—
I-c1	6.3	—
I-c2	0.9	—
G-Mu-a	−39.0	—
G-Mu-c	−33.6	—

^a^ Refers to G + Mu or corresponding I. ^b^ Refers to the related products.

**Table 3:** The comparison of antibacterial activities of nisin, Nisin@Ag and G-MuSH/Nisin@Ag expressed as μg/mL.

		Commercial Nisin	Nisin@Ag	G-MuSH/Nisin@Ag
*S. aureus*	MIC ^a^	281.25 ≡ [Nisin] = 7.03	66.67 ≡ [Nisin] = 1.39 and [Ag] = 5.62	178.75 ≡ [Nisin] = 2.79 and [Ag] = 11.25
	MBC ^b^	2250 ≡ [Nisin] = 56.25	535 ≡ [Nisin] = 11.15 and [Ag] = 45	NA ^c^
*E. coli*	MIC ^a^	NA^c^	66.67 ≡ [Nisin] = 1.39 and [Ag] = 5.62	178.75 ≡ [Nisin] = 2.79 and [Ag] = 11.25
	MBC ^b^	NA^c^	133 ≡ [Nisin] = 2.79 and [Ag] = 11.25	178.75 ≡ [Nisin] = 2.79 and [Ag] = 11.25
*P. aeruginosa*	MIC ^a^	NA^c^	133 ≡ [Nisin] = 2.79 and [Ag] = 11.25	357.5 ≡ [Nisin] = 5.57 and [Ag] = 22.5
	MBC ^b^	NA^c^	133 ≡ [Nisin] = 2.79 and [Ag] = 11.25	357.5 ≡ [Nisin] = 5.57 and [Ag] = 22.5

^a^ MIC: Minimal inhibitory concentration. ^b^ MBC: Minimal bactericidal concentration. ^c^ NA: No activity at the highest concentration tested.

## Data Availability

Not Applicable.

## Supplementary Materials

The following are available online at https://www.mdpi.com/article/10.3390/nano11071629/s1, Table S1: Free Energies (*G*), and the number of negative frequencies (*n*). All values are in Hartree; Cartesian Coordinates of DFT-Computed Stationary Points. Figure S1: Raman spectrum of pristine graphite; Figure S2: STEM image of G-MuSH.
