# Supplementary material for: Shedding Light on the Chemistry and the Properties of Münchnone Functionalized Graphene
Source: Nanomaterials (Basel). 2021 Jun 22;11(7):1629. doi: 10.3390/nano11071629 (PMC8307402; doi:10.3390/nano11071629)
Supplement: Supplementary file 1 [file nanomaterials-11-01629-s001.zip › nanomaterials-1252113-supplementary.pdf]

Supplementary Materials

Shedding Light on the Chemistry and the Properties of Münchnone Functionalized Graphene

Giulia Neri[1], Enza Fazio[2,*], Antonia Nostro[1], Placido Giuseppe Mineo[3], Angela Scala[1], Antonio Rescifina[4,*] and Anna Piperno[1,*]

1 Department of Chemical, Biological, Pharmaceutical and Environmental Sciences, University of Messina, V.le F. Stagno d'Alcontres 31, 98166 Messina, Italy; giulia.neri@unime.it (G.N.); anostro@unime.it (A.N.); ascala@unime.it (A.S.);
2 Department of Mathematical and Computational Sciences, Physics Science and Earth Science, University of Messina, Viale F. Stagno D'Alcontres 31, I-98166 Messina, Italy;
3 Department of Chemical Sciences, University of Catania, V.le A. Doria 6, 95125 Catania, Italy; gmineo@unict.it
4 Department of Drug and Health Sciences, University of Catania, V.le A. Doria, 95125 Catania, Italy;
* Correspondence: enfazio@unime.it (E.F.); arescifina@unict.it (A.R.); apiperno@unime.it (A.P.)

Table of Contents

Table S1. Free Energies (*G*) and number of negative frequencies (n). All values are in Hartree. S2

Cartesian Coordinates of DFT-Computed Stationary Points S2

Figure S1. Raman Spectrum of pristine graphite S26

Figure S2. STEM image of G-MuSH S26

Table 1. Free Energies (*G*) and number of negative frequencies (n). All values are in Hartree.

	G[a]	n
**Reagents**		
Mu	-630.540751	0
GF	-2679.457646	0
CO_2	-188.517805	0
H_2	-1.164864	0
**Transition Structures**		
TS-a1	-3309.973463	1
TS-a2	-3309.992484	1
TS-b	-3309.930210	1
TS-c1	-3309.978383	1
TS-c2	-3309.968172	1
TS-c3	-3309.965428	1
**Products**		
I-a1	-3310.023000	0
I-b	-3309.943500	0
I-c1	-3309.988377	0
I-c2	-3309.996900	0
G-Mu-a	-3120.377864	0
G-Mu-c	-3120.36923	0

[a]Computed at the (U)M06-2X/6-31G(d) level of theory.

Cartesian Coordinates of DFT-Computed Stationary Points

```
Mu
---------------------------------------------------------------------
 Center     Atomic      Atomic             Coordinates (Angstroms)
 Number     Number       Type             X           Y           Z
 ---------------------------------------------------------------------
      1          6           0       -3.692381   -1.375242    0.000160
      2          8           0       -3.098983    1.748885    0.000651
      3          8           0       -0.901275    1.041352    0.000320
      4          6           0       -2.328632    0.815813    0.000125
      5          6           0        1.999289   -1.277282    0.000352
      6          1           0        1.218129    2.027746   -0.000440
      7          1           0        3.678959    2.208919   -0.000482
      8          1           0        5.083739    0.154824   -0.000000
      9          1           0        3.986052   -2.072645    0.000573
     10          1           0        1.566612   -2.267824    0.000676
     11          1           0       -3.809516   -2.009073    0.888622
     12          1           0       -4.512833   -0.653337    0.000366
     13          6           0        3.385491   -1.168504    0.000325
     14          6           0        4.001348    0.078731    0.000015
     15          1           0       -3.809785   -2.008859   -0.888419
     16          6           0       -2.435683   -0.583907    0.000041
     17          6           0        3.213263    1.228585   -0.000253
     18          6           0        1.830373    1.133484   -0.000231
     19          6           0        1.197035   -0.123748    0.000047
     20          7           0       -1.150431   -1.103511   -0.000216
     21          6           0       -0.257369   -0.118365    0.000030
     22          6           0       -0.879583   -2.533431   -0.000610
     23          1           0       -0.319626   -2.812095   -0.895120
     24          1           0       -0.320247   -2.812728    0.894094
     25          1           0       -1.834204   -3.056206   -0.001123
```

```
GF
---------------------------------------------------------------------
 Center     Atomic      Atomic             Coordinates (Angstroms)
 Number     Number       Type             X           Y           Z
```

1	6	0	-7.071600	-0.000002	-0.000330
2	6	0	-7.784897	1.238381	-0.000347
3	6	0	-4.945691	1.231886	-0.000516
4	6	0	-5.669767	2.476694	0.000275
5	6	0	-2.820682	2.467002	-0.000872
6	6	0	-7.784891	-1.238386	-0.000345
7	6	0	-4.945693	-1.231886	-0.000515
8	6	0	-5.669761	-2.476695	0.000275
9	6	0	-2.820684	-2.467001	-0.000871
10	6	0	-5.656112	-0.000002	0.000151
11	6	0	-2.825477	0.000000	-0.002354
12	6	0	-3.537849	1.234562	0.001403
13	6	0	-0.703768	1.233435	-0.003335
14	6	0	-3.537844	-1.234562	0.001401
15	6	0	-0.703770	-1.233434	-0.003332
16	6	0	-1.415584	0.000001	0.003359
17	6	0	1.415585	0.000000	-0.003351
18	6	0	-3.551189	3.713952	0.000750
19	6	0	-0.694308	3.704951	-0.000731
20	6	0	-1.433898	4.953308	0.000479
21	6	0	1.433896	4.953308	-0.000452
22	6	0	-1.420761	2.468722	0.002073
23	6	0	1.420758	2.468723	-0.002062
24	6	0	0.694306	3.704950	0.000752
25	6	0	3.551188	3.713953	-0.000742
26	6	0	0.703768	1.233435	0.003351
27	6	0	3.537845	1.234563	-0.001405
28	6	0	2.820683	2.467003	0.000883
29	6	0	5.669759	2.476692	-0.000304
30	6	0	-3.551185	-3.713951	0.000748
31	6	0	-0.694308	-3.704949	-0.000730
32	6	0	-1.433895	-4.953306	0.000473
33	6	0	1.433896	-4.953306	-0.000451
34	6	0	-1.420755	-2.468722	0.002069
35	6	0	1.420756	-2.468721	-0.002054
36	6	0	0.703770	-1.233435	0.003350
37	6	0	3.537847	-1.234562	-0.001395
38	6	0	0.694311	-3.704949	0.000751
39	6	0	3.551185	-3.713951	-0.000730
40	6	0	2.820686	-2.467001	0.000890
41	6	0	5.669759	-2.476695	-0.000260
42	6	0	2.825476	0.000001	0.002370
43	6	0	5.656116	-0.000002	-0.000162
44	6	0	4.945691	1.231887	0.000509
45	6	0	7.784892	1.238388	0.000284
46	6	0	4.945691	-1.231888	0.000526
47	6	0	7.784898	-1.238387	0.000346
48	6	0	7.071597	0.000006	0.000307
49	6	0	-0.673297	6.190215	0.000384
50	1	0	-1.227590	7.124865	0.000453
51	6	0	-2.798606	4.938872	0.000999
52	1	0	-3.347338	5.878137	0.001102
53	6	0	-4.927152	3.696389	0.000767
54	1	0	-5.475229	4.635838	0.000879
55	6	0	-7.056597	2.453442	0.000066
56	1	0	-7.605665	3.392266	0.000111
57	6	0	-9.193371	1.208853	-0.000549
58	1	0	-9.738760	2.148346	-0.000581
59	6	0	-9.879281	-0.000007	-0.000718
60	1	0	-10.964846	-0.000000	-0.000861
61	6	0	-9.193376	-1.208856	-0.000547
62	1	0	-9.738755	-2.148356	-0.000575
63	6	0	-7.056600	-2.453441	0.000068
64	1	0	-7.605661	-3.392269	0.000115
65	6	0	-4.927153	-3.696387	0.000767
66	1	0	-5.475226	-4.635839	0.000879
67	6	0	-2.798605	-4.938869	0.000995
68	1	0	-3.347335	-5.878135	0.001097
69	6	0	-0.673296	-6.190212	0.000375

70	1	0	-1.227588	-7.124863	0.000442
71	6	0	0.673296	6.190215	-0.000344
72	1	0	1.227588	7.124865	-0.000407
73	6	0	2.798604	4.938872	-0.000977
74	1	0	3.347336	5.878137	-0.001074
75	6	0	4.927153	3.696388	-0.000776
76	1	0	5.475229	4.635838	-0.000881
77	6	0	7.056601	2.453440	-0.000131
78	1	0	7.605646	3.392278	-0.000201
79	1	0	3.347338	-5.878134	-0.001072
80	1	0	1.227591	-7.124862	-0.000418
81	6	0	2.798607	-4.938869	-0.000972
82	6	0	0.673297	-6.190212	-0.000353
83	6	0	9.193373	-1.208859	0.000536
84	1	0	9.738758	-2.148355	0.000608
85	6	0	7.056599	-2.453438	-0.000050
86	1	0	7.605658	-3.392268	-0.000087
87	6	0	4.927155	-3.696388	-0.000749
88	1	0	5.475227	-4.635840	-0.000861
89	6	0	9.879284	-0.000004	0.000651
90	6	0	9.193379	1.208849	0.000457
91	1	0	10.964849	0.000016	0.000749
92	1	0	9.738759	2.148348	0.000415

CO_2

Center Number	Atomic Number	Atomic Type	Coordinates (Angstroms) X	Y	Z
1	6	0	-1.090175	0.854643	0.000000
2	8	0	-2.252878	0.854643	-0.000000
3	8	0	0.072528	0.854643	0.000000

H_2

Center Number	Atomic Number	Atomic Type	Coordinates (Angstroms) X	Y	Z
1	1	0	-1.498230	0.706595	-0.028364
2	1	0	-2.235180	0.706595	-0.028364

TS-a1

Center Number	Atomic Number	Atomic Type	Coordinates (Angstroms) X	Y	Z
1	6	0	7.429944	0.172166	-0.255697
2	6	0	7.886871	1.500776	-0.515302
3	6	0	5.104624	0.941695	-0.449408
4	6	0	5.570845	2.278246	-0.712061
5	6	0	2.778772	1.717891	-0.623707
6	6	0	8.371517	-0.878145	-0.026821
7	6	0	5.586376	-1.426815	0.026530
8	6	0	6.539800	-2.481916	0.253519
9	6	0	3.745024	-3.033570	0.284455
10	6	0	6.042054	-0.104081	-0.227104
11	6	0	3.266426	-0.656984	-0.171225
12	6	0	3.723220	0.669707	-0.419536
13	6	0	0.944651	0.114063	-0.380382
14	6	0	4.206676	-1.706317	0.044651
15	6	0	1.429152	-2.266928	0.053766
16	6	0	1.884568	-0.936298	-0.171059
17	6	0	-0.886767	-1.509718	-0.218795
18	6	0	3.251241	3.056979	-0.886893
19	6	0	0.448148	2.507297	-0.775584
20	1	0	-1.626030	-7.564307	0.824986
21	6	0	-1.272877	-6.548446	0.661772
22	6	0	1.403767	1.450603	-0.595259
23	6	0	-1.376577	0.871054	-0.659737
24	6	0	-0.913599	2.220713	-0.818811
25	6	0	-3.695534	1.609749	-1.057854

26	6	0	-0.432410	-0.174238	-0.419345
27	6	0	-3.201356	-0.765846	-0.557239
28	6	0	-2.742042	0.569085	-0.755115
29	6	0	-5.524414	-0.019268	-0.923802
30	6	0	4.705098	-4.089180	0.513999
31	6	0	1.904265	-4.650295	0.505828
32	6	0	2.873009	-5.703494	0.746741
33	6	0	0.065173	-6.283266	0.701627
34	6	0	2.373727	-3.314609	0.279102
35	6	0	-0.408171	-3.889033	0.235174
36	6	0	0.051710	-2.553907	0.019839
37	6	0	-2.719479	-3.138589	-0.076354
38	6	0	0.544890	-4.931724	0.480833
39	6	0	-2.248247	-5.525360	0.400101
40	6	0	-1.777404	-4.175922	0.186203
41	6	0	-4.560481	-4.775161	0.069601
42	6	0	-2.264175	-1.804096	-0.284513
43	6	0	-5.029663	-2.393959	-0.423095
44	6	0	-4.575647	-1.063251	-0.634897
45	6	0	-7.349594	-1.649120	-0.778452
46	6	0	-4.095527	-3.429034	-0.143588
47	6	0	-6.868048	-4.026594	-0.278416
48	6	0	-6.412275	-2.689239	-0.492861
49	1	0	0.691333	-8.329527	1.124609
50	1	0	-3.948875	-6.809447	0.502079
51	6	0	2.271505	4.096067	-1.058265
52	1	0	2.628882	5.109479	-1.231730
53	6	0	4.603076	3.306234	-0.929162
54	1	0	4.956010	4.315988	-1.126184
55	6	0	6.934960	2.525856	-0.739865
56	1	0	7.289881	3.534602	-0.938191
57	6	0	9.273618	1.747339	-0.539186
58	1	0	9.624741	2.756201	-0.736767
59	6	0	10.182812	0.720327	-0.314273
60	1	0	11.247144	0.932567	-0.337263
61	6	0	9.746767	-0.574825	-0.061350
62	1	0	10.465173	-1.370971	0.112316
63	6	0	7.895015	-2.188096	0.224890
64	1	0	8.616754	-2.983266	0.397176
65	6	0	6.050357	-3.800661	0.498504
66	1	0	6.771270	-4.596382	0.671904
67	6	0	4.207243	-5.417043	0.749074
68	1	0	4.928571	-6.211315	0.928322
69	6	0	2.370005	-7.046310	0.975298
70	1	0	3.095539	-7.833274	1.162618
71	6	0	1.051424	-7.318426	0.954806
72	6	0	-3.596704	-5.792886	0.342266
73	6	0	-3.202536	2.952319	-1.213572
74	1	0	-3.927830	3.739878	-1.408370
75	6	0	-5.034719	1.307140	-1.131946
76	1	0	-5.749937	2.098615	-1.345051
77	6	0	-6.873984	-0.330982	-0.988440
78	1	0	-7.592410	0.455225	-1.209169
79	6	0	-8.719460	-1.971163	-0.842683
80	1	0	-9.434861	-1.183374	-1.061197
81	6	0	-9.154400	-3.274394	-0.632099
82	1	0	-10.214723	-3.500676	-0.686710
83	6	0	-8.249551	-4.291808	-0.353557
84	1	0	-8.599538	-5.307344	-0.191403
85	6	0	-5.919898	-5.041442	0.000197
86	1	0	-6.273158	-6.057482	0.160612
87	1	0	-2.089645	5.362636	-1.571635
88	1	0	0.306309	5.861908	-1.535790
89	6	0	-1.887032	3.274117	-1.051789
90	6	0	0.928704	3.867035	-0.968141
91	6	0	-1.433931	4.666286	-1.056530
92	6	0	-0.043512	4.957030	-1.046222
93	6	0	0.156541	5.808338	1.034611
94	6	0	-1.998381	5.471264	0.807866
95	6	0	-3.393436	5.130445	1.115941

96	7	0	-0.901187	5.106026	1.550845
97	6	0	-0.417728	7.021912	0.474763
98	8	0	-1.775720	6.773120	0.407287
99	8	0	0.087388	8.036953	0.070201
100	6	0	1.547615	5.747277	1.550793
101	1	0	1.999705	4.760809	1.406423
102	1	0	2.138861	6.483837	1.000559
103	1	0	1.592157	6.009427	2.614178
104	6	0	-3.781481	3.837401	1.496242
105	6	0	-4.383317	6.102938	0.910800
106	6	0	-5.723170	5.792592	1.104074
107	6	0	-5.125898	3.536060	1.695134
108	6	0	-6.100083	4.509472	1.499087
109	1	0	-4.089673	7.099297	0.600550
110	1	0	-6.476933	6.557672	0.946894
111	1	0	-3.049180	3.045437	1.599454
112	1	0	-5.407326	2.526850	1.978902
113	1	0	-7.148291	4.269665	1.647423
114	6	0	-0.755164	3.889975	2.329770
115	1	0	-1.565775	3.828705	3.057321
116	1	0	-0.751787	2.997791	1.695880
117	1	0	0.193512	3.947972	2.864259

TS-a2

Center Number	Atomic Number	Atomic Type	Coordinates (Angstroms) X	Y	Z
1	6	0	7.402909	-0.033570	-0.223092
2	6	0	7.884986	1.283524	-0.494682
3	6	0	5.091922	0.780612	-0.404261
4	6	0	5.584259	2.104078	-0.681531
5	6	0	2.780443	1.602869	-0.555197
6	6	0	8.324945	-1.100987	0.006016
7	6	0	5.530132	-1.594588	0.078070
8	6	0	6.463898	-2.667509	0.302825
9	6	0	3.659403	-3.165735	0.339547
10	6	0	6.010270	-0.282365	-0.183368
11	6	0	3.224477	-0.779936	-0.108197
12	6	0	3.705726	0.536286	-0.359438
13	6	0	0.916833	0.040016	-0.309457
14	6	0	4.145286	-1.847187	0.102490
15	6	0	1.358427	-2.355109	0.104501
16	6	0	1.837267	-1.030390	-0.107715
17	6	0	-0.940958	-1.558280	-0.186025
18	6	0	3.281481	2.923054	-0.838387
19	6	0	0.464877	2.455969	-0.666867
20	1	0	-1.789526	-7.609413	0.799744
21	6	0	-1.418333	-6.598216	0.648219
22	6	0	1.398871	1.371870	-0.509425
23	6	0	-1.386574	0.836449	-0.603353
24	6	0	-0.902186	2.184371	-0.723710
25	6	0	-3.691689	1.603337	-1.035212
26	6	0	-0.463535	-0.226909	-0.362742
27	6	0	-3.236805	-0.778893	-0.559364
28	6	0	-2.754136	0.551777	-0.729475
29	6	0	-5.542970	0.001629	-0.969863
30	6	0	4.599746	-4.239356	0.567246
31	6	0	1.789797	-4.749862	0.544451
32	6	0	2.738465	-5.820749	0.787698
33	6	0	-0.077273	-6.353898	0.710166
34	6	0	2.283246	-3.421001	0.328644
35	6	0	-0.506233	-3.949216	0.253270
36	6	0	-0.022842	-2.619451	0.055779
37	6	0	-2.799661	-3.162149	-0.091460
38	6	0	0.426539	-5.008771	0.503979
39	6	0	-2.373145	-5.558901	0.375868
40	6	0	-1.878438	-4.215193	0.180485
41	6	0	-4.666705	-4.772801	0.004171
42	6	0	-2.320805	-1.832771	-0.278066

43	6	0	-5.090997	-2.381977	-0.480617
44	6	0	-4.613450	-1.055810	-0.670270
45	6	0	-7.392689	-1.602315	-0.882411
46	6	0	-4.178017	-3.432123	-0.189025
47	6	0	-6.955122	-3.989211	-0.389173
48	6	0	-6.475821	-2.656822	-0.583768
49	1	0	0.510166	-8.411666	1.132886
50	1	0	-4.093825	-6.818506	0.435722
51	6	0	2.319380	3.981600	-1.012653
52	1	0	2.705270	4.972690	-1.236412
53	6	0	4.634165	3.148584	-0.902657
54	1	0	5.002879	4.149172	-1.116633
55	6	0	6.951953	2.325477	-0.721623
56	1	0	7.324728	3.325460	-0.931119
57	6	0	9.275898	1.502937	-0.529036
58	1	0	9.645401	2.503335	-0.735787
59	6	0	10.166042	0.459687	-0.302624
60	1	0	11.234178	0.650384	-0.333452
61	6	0	9.705575	-0.824804	-0.038741
62	1	0	10.409216	-1.633918	0.135455
63	6	0	7.824411	-2.400121	0.266541
64	1	0	8.531442	-3.208564	0.437878
65	6	0	5.950128	-3.976240	0.551002
66	1	0	6.656098	-4.785490	0.723040
67	6	0	4.077455	-5.558279	0.798314
68	1	0	4.783629	-6.365842	0.978311
69	6	0	2.211156	-7.155685	1.007055
70	1	0	2.921745	-7.955069	1.198884
71	6	0	0.888690	-7.406017	0.971161
72	6	0	-3.723842	-5.806206	0.289909
73	6	0	-3.175723	2.944212	-1.151771
74	1	0	-3.888417	3.748525	-1.324930
75	6	0	-5.030576	1.324067	-1.148931
76	1	0	-5.729514	2.127235	-1.370750
77	6	0	-6.894301	-0.289225	-1.070049
78	1	0	-7.596277	0.509431	-1.298434
79	6	0	-8.765019	-1.904302	-0.979831
80	1	0	-9.464231	-1.104890	-1.208067
81	6	0	-9.222494	-3.202724	-0.788397
82	1	0	-10.284313	-3.413845	-0.868076
83	6	0	-8.338115	-4.234347	-0.497013
84	1	0	-8.705518	-5.245980	-0.349774
85	6	0	-6.027822	-5.019104	-0.096671
86	1	0	-6.398723	-6.031059	0.048916
87	1	0	-2.036428	5.302127	-1.501263
88	1	0	0.273808	5.548841	-1.859495
89	6	0	-1.866499	3.236192	-0.958870
90	6	0	0.983511	3.798563	-0.864145
91	6	0	-1.449172	4.679729	-0.822865
92	6	0	0.065165	5.005097	-0.933586
93	6	0	0.242021	6.012876	0.247874
94	6	0	-1.780178	5.142911	0.593895
95	6	0	-3.100875	4.970390	1.236897
96	7	0	-0.698116	5.475731	1.240774
97	6	0	-0.492180	7.355687	-0.276059
98	8	0	-1.746835	7.294144	-0.173138
99	8	0	0.253736	8.202193	-0.754937
100	6	0	1.619347	6.320767	0.783761
101	1	0	2.143667	5.436754	1.154756
102	1	0	2.193445	6.790123	-0.016333
103	1	0	1.548329	7.068043	1.579071
104	6	0	-3.413737	3.757444	1.860388
105	6	0	-4.036447	6.006209	1.186097
106	6	0	-5.290287	5.815630	1.762525
107	6	0	-4.667671	3.581976	2.434971
108	6	0	-5.606902	4.610394	2.383790
109	1	0	-3.762218	6.934517	0.696974
110	1	0	-6.022043	6.616374	1.724486
111	1	0	-2.683415	2.952277	1.877205
112	1	0	-4.912486	2.638803	2.912515

113	1	0	-6.587310	4.469575	2.828184
114	6	0	-0.729070	6.033105	2.585304
115	1	0	-0.929046	7.108118	2.514850
116	1	0	-1.520996	5.550063	3.156244
117	1	0	0.233062	5.859961	3.067184

TS-b

Center Number	Atomic Number	Atomic Type	Coordinates (Angstroms) X	Y	Z
1	6	0	-7.322062	0.171817	-0.562750
2	6	0	-8.016820	-1.068987	-0.722598
3	6	0	-5.174198	-1.039496	-0.510903
4	6	0	-5.886230	-2.284042	-0.705046
5	6	0	-3.037573	-2.260710	-0.458620
6	6	0	-8.053598	1.397507	-0.506650
7	6	0	-5.224438	1.415401	-0.328962
8	6	0	-5.963399	2.648488	-0.282077
9	6	0	-3.121571	2.662437	-0.125177
10	6	0	-5.911442	0.185893	-0.464143
11	6	0	-3.080127	0.205669	-0.236104
12	6	0	-3.785536	-1.034115	-0.379933
13	1	0	0.859733	7.369784	0.036507
14	6	0	-3.812131	1.431346	-0.242320
15	6	0	-0.995182	1.442998	-0.070361
16	6	0	-1.686041	0.217381	-0.070718
17	6	0	1.164172	0.261754	-0.228421
18	6	0	-3.753604	-3.493639	-0.718922
19	6	0	-0.915235	-3.456468	-0.545960
20	6	0	-1.630914	-4.693783	-0.788909
21	6	0	1.220103	-4.649589	-0.896085
22	6	0	-1.659020	-2.264616	-0.302117
23	6	0	1.201165	-2.212354	-0.454414
24	6	0	0.484438	-3.435405	-0.605314
25	6	0	3.305297	-3.381522	-0.983182
26	6	0	0.317658	6.428378	0.009409
27	6	0	3.272505	-0.915050	-0.700617
28	6	0	2.565312	-2.165732	-0.714134
29	6	0	5.398047	-2.105273	-1.071288
30	6	0	-3.867814	3.901164	-0.087022
31	6	0	-1.011347	3.921644	-0.048537
32	6	0	-1.768038	5.159697	0.016557
33	6	0	1.091651	5.206459	-0.112312
34	6	0	-1.718852	2.672067	-0.079395
35	6	0	1.115170	2.718034	-0.215108
36	6	0	0.437185	1.467451	-0.147354
37	6	0	3.227186	1.550364	-0.573142
38	6	0	0.372998	3.944233	-0.112971
39	6	0	3.216206	4.017485	-0.394172
40	6	0	2.509475	2.755806	-0.387756
41	6	0	5.336471	2.833009	-0.713598
42	6	0	2.538198	0.301224	-0.519527
43	6	0	5.352838	0.369317	-0.880366
44	6	0	4.654488	-0.879106	-0.883628
45	6	0	7.489211	-0.827027	-1.165872
46	6	0	4.633039	1.578295	-0.731987
47	6	0	7.456315	1.644441	-0.993163
48	6	0	6.760566	0.396066	-1.013360
49	6	0	-0.863923	-5.909358	-0.983948
50	1	0	-1.413388	-6.838293	-1.109238
51	6	0	-2.993813	-4.698179	-0.846915
52	1	0	-3.526051	-5.631312	-1.016165
53	6	0	-5.125697	-3.485377	-0.812256
54	1	0	-5.655180	-4.420998	-0.976688
55	6	0	-7.264562	-2.273034	-0.798650
56	1	0	-7.796256	-3.210953	-0.941949
57	6	0	-9.415125	-1.055094	-0.812259
58	1	0	-9.944770	-1.995762	-0.934150
59	6	0	-10.124374	0.145955	-0.748386

60	1	0	-11.207536	0.131082	-0.819235
61	6	0	-9.463688	1.352783	-0.599732
62	1	0	-10.021600	2.283820	-0.554541
63	6	0	-7.348904	2.612789	-0.364450
64	1	0	-7.909538	3.543884	-0.323814
65	6	0	-5.239869	3.872292	-0.155273
66	1	0	-5.798913	4.804748	-0.124808
67	6	0	-3.131339	5.131089	0.008417
68	1	0	-3.690616	6.063106	0.051757
69	6	0	-1.026719	6.406499	0.069971
70	1	0	-1.594664	7.329732	0.146841
71	6	0	0.479457	-5.889546	-1.032033
72	1	0	1.045890	-6.801851	-1.197840
73	6	0	2.576972	-4.611143	-1.048008
74	1	0	3.125569	-5.529228	-1.245805
75	6	0	4.672597	-3.332159	-1.127654
76	1	0	5.225862	-4.253885	-1.293906
77	6	0	6.772837	-2.054821	-1.199572
78	1	0	7.330143	-2.978682	-1.337505
79	6	0	8.884379	-0.772724	-1.288871
80	1	0	9.439644	-1.699070	-1.406368
81	6	0	9.557123	0.450388	-1.265029
82	1	0	10.638297	0.466871	-1.362860
83	6	0	8.864478	1.639837	-1.121180
84	1	0	9.395106	2.587632	-1.105638
85	6	0	6.719244	2.839367	-0.842839
86	1	0	7.252078	3.787333	-0.825479
87	6	0	4.581671	4.033473	-0.549628
88	1	0	5.111171	4.983563	-0.548093
89	6	0	2.449619	5.222522	-0.236521
90	1	0	2.979569	6.172562	-0.235918
91	6	0	0.535599	-1.025600	0.104937
92	6	0	-0.976852	-1.052353	0.230404
93	6	0	-1.198897	-1.127937	2.114882
94	6	0	0.978927	-1.229660	2.031845
95	6	0	2.412943	-1.040964	2.314716
96	7	0	-0.045881	-0.452971	2.547230
97	6	0	-0.802481	-2.553802	2.303012
98	8	0	0.561387	-2.558403	2.197210
99	8	0	-1.464403	-3.539202	2.448827
100	6	0	-2.542598	-0.735829	2.649539
101	1	0	-2.811696	0.289503	2.386986
102	1	0	-3.295878	-1.416628	2.247475
103	1	0	-2.546886	-0.842352	3.739721
104	6	0	3.000018	0.229587	2.374171
105	6	0	3.234138	-2.176776	2.370006
106	6	0	4.613059	-2.039508	2.464327
107	6	0	4.380724	0.359398	2.477992
108	6	0	5.191292	-0.772530	2.510246
109	1	0	2.784609	-3.161315	2.304809
110	1	0	5.239257	-2.925891	2.480250
111	1	0	2.395505	1.125562	2.274134
112	1	0	4.823560	1.350750	2.493577
113	1	0	6.270892	-0.666046	2.555678
114	6	0	0.018463	0.871699	3.136124
115	1	0	0.782373	0.873845	3.917522
116	1	0	0.232400	1.665914	2.415816
117	1	0	-0.948707	1.073048	3.599919

TS-c1

Center Number	Atomic Number	Atomic Type	Coordinates (Angstroms) X	Y	Z
1	6	0	8.695305	-0.178143	0.614171
2	6	0	9.443828	1.033774	0.789492
3	6	0	6.627100	1.136002	0.377405
4	6	0	7.385590	2.347110	0.553904
5	6	0	4.562387	2.450000	0.141476

6	6	0	9.364545	-1.447142	0.645481
7	6	0	6.549234	-1.323290	0.234641
8	6	0	7.229133	-2.592823	0.267198
9	6	0	4.405263	-2.466322	-0.144774
10	6	0	7.292517	-0.121991	0.410344
11	6	0	4.488253	-0.008471	-0.000079
12	6	0	5.233528	1.195061	0.178187
13	6	0	2.426485	1.306870	-0.231378
14	6	0	5.154690	-1.269797	0.034735
15	6	0	2.347859	-1.152859	-0.375772
16	6	0	3.091429	0.047934	-0.193635
17	6	0	0.287013	0.162004	-0.598736
18	6	0	5.322625	3.660473	0.316576
19	6	0	2.498521	3.767488	-0.092856
20	6	0	3.267456	4.981061	0.079965
21	6	0	0.429598	5.097032	-0.325517
22	6	0	3.173769	2.508360	-0.054500
23	6	0	0.364978	2.624176	-0.456507
24	6	0	1.118000	3.824405	-0.287025
25	6	0	-1.707937	3.949914	-0.677368
26	6	0	1.030787	1.364493	-0.420106
27	6	0	-1.773469	1.479249	-0.805518
28	6	0	-1.030081	2.679297	-0.642651
29	6	0	-3.845418	2.801056	-0.993015
30	6	0	5.086687	-3.734816	-0.113707
31	6	0	2.261892	-3.611828	-0.524820
32	6	0	2.950956	-4.883406	-0.496571
33	6	0	0.113618	-4.769102	-0.909431
34	6	0	3.016228	-2.412337	-0.342173
35	6	0	0.207723	-2.299192	-0.750340
36	6	0	0.952102	-1.098331	-0.566983
37	6	0	-1.851575	-0.985431	-0.966089
38	6	0	0.881143	-3.556756	-0.722398
39	6	0	-1.942144	-3.453243	-1.132612
40	6	0	-1.186807	-2.242051	-0.945974
41	6	0	-3.994322	-2.137101	-1.351872
42	6	0	-1.108568	0.217917	-0.782909
43	6	0	-3.920523	0.331071	-1.145395
44	6	0	-3.175288	1.534037	-0.973459
45	6	0	-5.999089	1.646506	-1.201362
46	6	0	-3.250852	-0.929443	-1.153088
47	6	0	-6.055208	-0.826747	-1.490878
48	6	0	-5.326853	0.384456	-1.274266
49	6	0	2.556400	6.244388	0.036700
50	1	0	3.134993	7.155798	0.163052
51	6	0	4.619717	4.910047	0.275418
52	1	0	5.192071	5.826698	0.403962
53	6	0	6.686893	3.588325	0.514427
54	1	0	7.255979	4.506484	0.644625
55	6	0	8.757781	2.270783	0.753049
56	1	0	9.327396	3.187775	0.884791
57	6	0	10.836070	0.947509	0.991208
58	1	0	11.405451	1.862713	1.123847
59	6	0	11.468979	-0.288634	1.019128
60	1	0	12.543381	-0.332014	1.175894
61	6	0	10.759385	-1.470438	0.851032
62	1	0	11.269942	-2.428681	0.875402
63	6	0	8.602804	-2.625981	0.469095
64	1	0	9.113171	-3.585925	0.492281
65	6	0	6.452048	-3.773592	0.086673
66	1	0	6.960403	-4.735239	0.107688
67	6	0	4.304349	-4.922775	-0.297781
68	1	0	4.815588	-5.883303	-0.278545
69	6	0	2.160524	-6.084539	-0.685627
70	1	0	2.678653	-7.039876	-0.667506
71	6	0	1.223309	6.300216	-0.154871
72	1	0	0.707477	7.256180	-0.185471
73	6	0	-0.925853	5.138738	-0.515093
74	1	0	-1.435529	6.099249	-0.542977
75	6	0	-3.083364	3.983197	-0.853806

76	1	0	-3.594656	4.943010	-0.875685
77	6	0	-5.251122	2.816905	-1.111188
78	1	0	-5.759563	3.778979	-1.129793
79	1	0	-1.809756	-5.616406	-1.249071
80	1	0	0.252181	-6.943130	-1.022669
81	6	0	-1.240448	-4.700623	-1.105384
82	6	0	0.827602	-6.032218	-0.880772
83	6	0	-7.482726	-0.733064	-1.645097
84	1	0	-8.033264	-1.637714	-1.891456
85	6	0	-5.390117	-2.043400	-1.540567
86	1	0	-5.961312	-2.955083	-1.704206
87	6	0	-3.314804	-3.374560	-1.331694
88	1	0	-3.885495	-4.288442	-1.483072
89	6	0	-8.141163	0.447844	-1.513813
90	6	0	-7.451391	1.654303	-1.110208
91	1	0	-9.219787	0.489227	-1.635128
92	1	0	-7.953781	2.597641	-1.324775
93	6	0	-7.822775	1.831500	0.902851
94	6	0	-7.674427	-0.296376	1.571402
95	6	0	-7.191347	-1.618766	1.900190
96	7	0	-6.999993	0.836393	1.484546
97	6	0	-9.160307	1.262159	0.966214
98	8	0	-8.968762	-0.099613	1.312040
99	8	0	-10.241202	1.693257	0.690456
100	6	0	-7.613790	3.274355	1.238988
101	1	0	-6.650659	3.634874	0.867302
102	1	0	-8.414927	3.850214	0.768823
103	1	0	-7.662249	3.451489	2.319321
104	6	0	-5.869299	-1.993403	1.610162
105	6	0	-8.089887	-2.555724	2.437307
106	6	0	-7.652431	-3.840544	2.715007
107	6	0	-5.447492	-3.290038	1.880930
108	6	0	-6.332607	-4.208304	2.439704
109	1	0	-9.116346	-2.262222	2.631045
110	1	0	-8.340640	-4.562440	3.141639
111	1	0	-5.185660	-1.298391	1.133249
112	1	0	-4.431243	-3.579686	1.632747
113	1	0	-5.999275	-5.219021	2.652259
114	6	0	-5.642189	1.092195	1.965454
115	1	0	-5.401712	0.360096	2.735949
116	1	0	-4.915897	1.038968	1.152487
117	1	0	-5.627856	2.092642	2.399098

TS-c2

Center Number	Atomic Number	Atomic Type	Coordinates (Angstroms) X	Y	Z
1	6	0	-9.051568	0.208307	0.507500
2	6	0	-9.799429	-1.008301	0.616210
3	6	0	-6.975307	-1.092077	0.311575
4	6	0	-7.732028	-2.310438	0.418379
5	6	0	-4.899966	-2.392277	0.114026
6	6	0	-9.725325	1.472000	0.551499
7	6	0	-6.898470	1.370831	0.247420
8	6	0	-7.582911	2.637023	0.289776
9	6	0	-4.748468	2.533783	-0.013185
10	6	0	-7.644193	0.161886	0.355544
11	6	0	-4.826776	0.070061	0.056849
12	6	0	-5.573405	-1.141096	0.159730
13	6	0	-2.756103	-1.230795	-0.135151
14	6	0	-5.499997	1.327621	0.095994
15	6	0	-2.678467	1.234062	-0.198577
16	6	0	-3.426496	0.023938	-0.096212
17	6	0	-0.607620	-0.067474	-0.383339
18	6	0	-5.660755	-3.611804	0.217476
19	6	0	-2.822474	-3.695251	-0.088049
20	6	0	-3.592893	-4.917497	0.013518
21	6	0	-0.740843	-5.009393	-0.295944
22	6	0	-3.503345	-2.439891	-0.037609

23	6	0	-0.683969	-2.531716	-0.332819
24	6	0	-1.436987	-3.740322	-0.237873
25	6	0	1.400774	-3.840562	-0.538889
26	6	0	-1.353774	-1.277815	-0.286448
27	6	0	1.459687	-1.369751	-0.566513
28	6	0	0.717988	-2.575467	-0.479041
29	6	0	3.537166	-2.671043	-0.756493
30	6	0	-5.436906	3.800499	0.025892
31	6	0	-2.598146	3.699294	-0.277869
32	6	0	-3.295362	4.968886	-0.240797
33	6	0	-0.445273	4.876346	-0.550523
34	6	0	-3.355489	2.490198	-0.164256
35	6	0	-0.530799	2.398808	-0.460446
36	6	0	-1.280910	1.189060	-0.350232
37	6	0	1.541685	1.097771	-0.637634
38	6	0	-1.214521	3.654525	-0.427720
39	6	0	1.622133	3.572003	-0.736726
40	6	0	0.864508	2.351771	-0.610471
41	6	0	3.688039	2.270186	-0.921169
42	6	0	0.791451	-0.113927	-0.527229
43	6	0	3.617738	-0.208382	-0.793183
44	6	0	2.868543	-1.414412	-0.697395
45	6	0	5.692576	-1.513653	-0.877570
46	6	0	2.939766	1.050187	-0.779913
47	6	0	5.757023	0.968847	-1.103723
48	6	0	5.024245	-0.259591	-0.902077
49	6	0	-2.873663	-6.176719	-0.047124
50	1	0	-3.451837	-7.093837	0.028843
51	6	0	-4.950478	-4.858464	0.160780
52	1	0	-5.523440	-5.780213	0.237949
53	6	0	-7.030992	-3.549656	0.365761
54	1	0	-7.602419	-4.471878	0.445222
55	6	0	-9.113277	-2.242019	0.567724
56	1	0	-9.685430	-3.163233	0.649502
57	6	0	-11.201466	-0.930628	0.767528
58	1	0	-11.771765	-1.851262	0.850506
59	6	0	-11.841200	0.299542	0.809460
60	1	0	-12.920356	0.336407	0.926393
61	6	0	-11.125156	1.487807	0.703915
62	1	0	-11.639259	2.443971	0.737917
63	6	0	-8.962720	2.659907	0.439642
64	1	0	-9.478531	3.616758	0.472489
65	6	0	-6.807044	3.828269	0.174253
66	1	0	-7.321992	4.786000	0.204747
67	6	0	-4.653538	4.998225	-0.093556
68	1	0	-5.170528	5.955187	-0.065180
69	6	0	-2.503958	6.179128	-0.364696
70	1	0	-3.027284	7.131226	-0.336737
71	6	0	-1.534904	-6.220555	-0.193322
72	1	0	-1.013687	-7.173031	-0.238141
73	6	0	0.618727	-5.038754	-0.443202
74	1	0	1.134237	-5.995520	-0.489101
75	6	0	2.779368	-3.861769	-0.681316
76	1	0	3.296365	-4.817650	-0.728218
77	6	0	4.948284	-2.677469	-0.859841
78	1	0	5.454434	-3.639602	-0.907069
79	1	0	1.483537	5.738741	-0.795674
80	1	0	-0.588362	7.052446	-0.603829
81	6	0	0.912327	4.817930	-0.699930
82	6	0	-1.165394	6.136294	-0.510851
83	6	0	7.139456	0.894500	-1.354237
84	1	0	7.657891	1.804867	-1.649013
85	6	0	5.071848	2.193112	-1.100898
86	1	0	5.638617	3.110253	-1.251138
87	6	0	2.994053	3.506776	-0.892552
88	1	0	3.563852	4.427101	-0.999842
89	6	0	7.883791	-0.270895	-1.192006
90	6	0	7.201714	-1.579947	-0.858884
91	1	0	8.845665	-0.321225	-1.694482
92	1	0	7.532024	-2.378870	-1.533505

93	6	0	7.788401	-2.037405	0.603831
94	6	0	8.840668	-0.112550	0.744336
95	6	0	9.274897	1.249066	1.042228
96	7	0	7.881587	-0.832841	1.398567
97	6	0	9.261269	-2.245660	0.307246
98	8	0	9.843174	-1.005141	0.384916
99	8	0	9.849694	-3.232589	-0.013589
100	6	0	7.149919	-3.233165	1.266112
101	1	0	6.078092	-3.091327	1.415534
102	1	0	7.304302	-4.111637	0.634075
103	1	0	7.631884	-3.421870	2.229275
104	6	0	8.345458	2.286113	1.223752
105	6	0	10.646808	1.533923	1.094455
106	6	0	11.078384	2.828897	1.352439
107	6	0	8.789065	3.577231	1.479437
108	6	0	10.153811	3.852518	1.549505
109	1	0	11.363467	0.735489	0.938669
110	1	0	12.141962	3.038881	1.400234
111	1	0	7.281759	2.091199	1.133868
112	1	0	8.064699	4.373332	1.618413
113	1	0	10.494723	4.863072	1.750092
114	6	0	6.755778	-0.285634	2.134469
115	1	0	5.984926	0.146554	1.489783
116	1	0	6.313508	-1.097059	2.715755
117	1	0	7.127414	0.471019	2.827459

TS-c3

Center Number	Atomic Number	Atomic Type	Coordinates (Angstroms) X	Y	Z
1	6	0	-9.137618	0.084000	0.462314
2	6	0	-9.857536	-1.146260	0.553137
3	6	0	-7.035234	-1.168064	0.282702
4	6	0	-7.763433	-2.405013	0.372746
5	6	0	-4.931717	-2.421840	0.100954
6	6	0	-9.837523	1.329069	0.506679
7	6	0	-7.010373	1.295324	0.236221
8	6	0	-7.721771	2.547411	0.280017
9	6	0	-4.886479	2.511030	0.008523
10	6	0	-7.729771	0.069468	0.328602
11	6	0	-4.911727	0.042966	0.059284
12	6	0	-5.630216	-1.184638	0.152207
13	6	0	-2.813281	-1.209287	-0.116718
14	6	0	-5.611624	1.284405	0.106553
15	6	0	-2.787690	1.257516	-0.163592
16	6	0	-3.508713	0.029526	-0.063465
17	6	0	-0.688580	0.004025	-0.328583
18	6	0	-5.665953	-3.661590	0.188734
19	6	0	-2.823720	-3.677163	-0.085628
20	6	0	-3.568023	-4.918558	-0.001388
21	6	0	-0.712340	-4.942224	-0.280691
22	6	0	-3.532500	-2.436448	-0.027245
23	6	0	-0.710264	-2.460690	-0.296329
24	6	0	-1.437610	-3.688753	-0.215943
25	6	0	1.403510	-3.720428	-0.491275
26	6	0	-1.406611	-1.222618	-0.236550
27	6	0	1.408577	-1.248107	-0.491644
28	6	0	0.692806	-2.470846	-0.421041
29	6	0	3.514155	-2.499208	-0.678616
30	6	0	-5.604540	3.765003	0.050173
31	6	0	-2.761824	3.728906	-0.223739
32	6	0	-3.489347	4.983966	-0.185595
33	6	0	-0.636994	4.957169	-0.465547
34	6	0	-3.494140	2.499840	-0.118746
35	6	0	-0.662766	2.473195	-0.390597
36	6	0	-1.389811	1.244387	-0.282337
37	6	0	1.442334	1.218689	-0.545689
38	6	0	-1.379671	3.716013	-0.354103
39	6	0	1.461584	3.698759	-0.635403

40	6	0	0.727929	2.458768	-0.516897
41	6	0	3.565611	2.444768	-0.800504
42	6	0	0.713875	-0.010310	-0.442162
43	6	0	3.549248	-0.039155	-0.675815
44	6	0	2.821641	-1.259291	-0.603514
45	6	0	5.643872	-1.299702	-0.782377
46	6	0	2.835300	1.201892	-0.663998
47	6	0	5.685705	1.194663	-0.940339
48	6	0	4.958365	-0.065077	-0.762531
49	6	0	-2.819507	-6.161209	-0.064753
50	1	0	-3.377454	-7.091584	-0.003094
51	6	0	-4.927096	-4.892511	0.130968
52	1	0	-5.480347	-5.826905	0.193746
53	6	0	-7.037653	-3.630664	0.320699
54	1	0	-7.591053	-4.564556	0.386479
55	6	0	-9.147506	-2.366577	0.505370
56	1	0	-9.702077	-3.299616	0.573963
57	6	0	-11.262972	-1.100659	0.687694
58	1	0	-11.813639	-2.034523	0.757430
59	6	0	-11.933649	0.112265	0.730559
60	1	0	-13.013955	0.125483	0.834426
61	6	0	-11.236791	1.315580	0.641630
62	1	0	-11.771586	2.260567	0.675940
63	6	0	-9.101262	2.538015	0.412798
64	1	0	-9.640573	3.481944	0.446054
65	6	0	-6.974114	3.760367	0.182317
66	1	0	-7.513150	4.704490	0.212982
67	6	0	-4.847677	4.982547	-0.052886
68	1	0	-5.387552	5.926428	-0.025044
69	6	0	-2.725074	6.213659	-0.294503
70	1	0	-3.270828	7.152861	-0.267247
71	6	0	-1.479153	-6.172199	-0.197560
72	1	0	-0.935271	-7.111707	-0.244939
73	6	0	0.647293	-4.938428	-0.415438
74	1	0	1.186118	-5.882026	-0.465579
75	6	0	2.779445	-3.710257	-0.624989
76	1	0	3.319832	-4.652447	-0.681179
77	6	0	4.919349	-2.478950	-0.789936
78	1	0	5.443264	-3.427540	-0.873155
79	1	0	1.272385	5.863431	-0.687372
80	1	0	-0.827362	7.129629	-0.509691
81	6	0	0.720500	4.929891	-0.601677
82	6	0	-1.385251	6.200731	-0.427584
83	6	0	7.034111	1.197118	-1.132720
84	1	0	7.541331	2.137936	-1.330835
85	6	0	4.933236	2.415117	-0.953323
86	1	0	5.477704	3.348352	-1.078588
87	6	0	2.828530	3.669770	-0.776826
88	1	0	3.377522	4.603717	-0.873771
89	6	0	7.897515	-0.014252	-0.967643
90	6	0	7.160916	-1.380715	-0.838190
91	1	0	8.685485	-0.049630	-1.725198
92	1	0	7.426597	-2.033641	-1.675057
93	6	0	7.838708	-1.981052	0.436059
94	6	0	8.571092	0.130937	0.386637
95	6	0	9.384244	1.301441	0.780962
96	7	0	8.145530	-0.774846	1.220068
97	6	0	9.288150	-2.464100	-0.095764
98	8	0	10.092397	-1.507775	-0.245142
99	8	0	9.374689	-3.662371	-0.344060
100	6	0	7.141058	-3.072053	1.211285
101	1	0	6.146177	-2.781052	1.558591
102	1	0	7.072095	-3.949350	0.567172
103	1	0	7.755450	-3.375733	2.063465
104	6	0	8.764166	2.434284	1.318380
105	6	0	10.769064	1.265865	0.599173
106	6	0	11.527982	2.376502	0.958858
107	6	0	9.535935	3.534724	1.678157
108	6	0	10.916978	3.506611	1.496707
109	1	0	11.217691	0.368126	0.187394

110	1	0	12.604180	2.355229	0.819520
111	1	0	7.685090	2.447767	1.447730
112	1	0	9.057356	4.413597	2.098302
113	1	0	11.516848	4.367261	1.775765
114	6	0	8.666187	-0.907141	2.572952
115	1	0	9.571596	-1.523240	2.541335
116	1	0	8.908701	0.080125	2.963814
117	1	0	7.912858	-1.379163	3.203799

I-a1

Center Number	Atomic Number	Atomic Type	Coordinates (Angstroms) X	Y	Z
1	6	0	7.436622	0.191119	-0.271145
2	6	0	7.882712	1.521085	-0.542282
3	6	0	5.103972	0.947479	-0.429361
4	6	0	5.560224	2.284450	-0.704689
5	6	0	2.770613	1.710887	-0.557954
6	6	0	8.387447	-0.853519	-0.053614
7	6	0	5.606527	-1.417483	0.042135
8	6	0	6.569014	-2.466921	0.256191
9	6	0	3.778536	-3.035851	0.317862
10	6	0	6.050974	-0.092785	-0.219977
11	6	0	3.279540	-0.661180	-0.122157
12	6	0	3.724733	0.667863	-0.373681
13	6	0	0.950337	0.099059	-0.302434
14	6	0	4.228871	-1.705031	0.078591
15	6	0	1.455963	-2.283610	0.104253
16	6	0	1.899341	-0.947154	-0.109369
17	6	0	-0.864964	-1.544041	-0.168827
18	6	0	3.235636	3.045914	-0.835489
19	6	0	0.432279	2.499978	-0.647777
20	1	0	-1.553072	-7.615514	0.820402
21	6	0	-1.208656	-6.595231	0.666442
22	6	0	1.395875	1.442650	-0.502055
23	6	0	-1.374171	0.839973	-0.583731
24	6	0	-0.928384	2.202003	-0.701264
25	6	0	-3.693490	1.540579	-1.029567
26	6	0	-0.423402	-0.200180	-0.345635
27	6	0	-3.180629	-0.825302	-0.534788
28	6	0	-2.732406	0.516196	-0.711267
29	6	0	-5.506165	-0.103570	-0.940294
30	6	0	4.747562	-4.085711	0.535593
31	6	0	1.951236	-4.666801	0.537838
32	6	0	2.928587	-5.713643	0.771835
33	6	0	0.126307	-6.317100	0.718093
34	6	0	2.409237	-3.325831	0.319405
35	6	0	-0.366657	-3.923812	0.265925
36	6	0	0.081311	-2.581902	0.066712
37	6	0	-2.681326	-3.195398	-0.063946
38	6	0	0.594495	-4.959659	0.508779
39	6	0	-2.191459	-5.580404	0.401186
40	6	0	-1.732213	-4.224615	0.203007
41	6	0	-4.506472	-4.852583	0.042963
42	6	0	-2.237527	-1.854418	-0.253040
43	6	0	-4.993397	-2.473876	-0.443798
44	6	0	-4.550152	-1.136895	-0.639785
45	6	0	-7.315347	-1.752822	-0.836166
46	6	0	-4.052948	-3.500191	-0.154210
47	6	0	-6.815922	-4.127047	-0.339535
48	6	0	-6.371273	-2.783586	-0.539369
49	1	0	0.768628	-8.359485	1.135195
50	1	0	-3.879664	-6.882903	0.472256
51	6	0	2.243258	4.080320	-0.985860
52	1	0	2.599601	5.088889	-1.184172
53	6	0	4.581695	3.305779	-0.910060
54	1	0	4.923751	4.316945	-1.118780
55	6	0	6.921611	2.540027	-0.756122
56	1	0	7.267501	3.549981	-0.963954

57	6	0	9.267461	1.775063	-0.588225
58	1	0	9.610046	2.785120	-0.794433
59	6	0	10.185463	0.753711	-0.373580
60	1	0	11.248176	0.971544	-0.413254
61	6	0	9.760298	-0.542865	-0.109897
62	1	0	10.485530	-1.334596	0.055352
63	6	0	7.921966	-2.165420	0.208004
64	1	0	8.650599	-2.956255	0.370983
65	6	0	6.090705	-3.788622	0.506943
66	1	0	6.818457	-4.580034	0.671219
67	6	0	4.260660	-5.417563	0.770290
68	1	0	4.988517	-6.207270	0.943156
69	6	0	2.436678	-7.061395	0.995194
70	1	0	3.168606	-7.842872	1.180578
71	6	0	1.120572	-7.344668	0.970597
72	6	0	-3.536113	-5.861687	0.323868
73	6	0	-3.210236	2.890072	-1.168406
74	1	0	-3.946345	3.665828	-1.370809
75	6	0	-5.026484	1.228965	-1.133601
76	1	0	-5.744908	2.014856	-1.355647
77	6	0	-6.850616	-0.428588	-1.031430
78	1	0	-7.573331	0.351350	-1.260037
79	6	0	-8.680293	-2.089210	-0.925749
80	1	0	-9.400372	-1.308054	-1.152483
81	6	0	-9.104311	-3.398112	-0.728856
82	1	0	-10.160965	-3.635824	-0.802456
83	6	0	-8.192946	-4.406762	-0.439783
84	1	0	-8.534428	-5.426865	-0.288507
85	6	0	-5.861619	-5.133008	-0.050261
86	1	0	-6.206459	-6.153668	0.098689
87	1	0	-2.046852	5.223197	-1.694126
88	1	0	0.242075	5.694189	-1.654413
89	6	0	-1.915831	3.231036	-0.950154
90	6	0	0.914707	3.853941	-0.831687
91	6	0	-1.553428	4.690992	-0.876301
92	6	0	-0.032819	5.030720	-0.827882
93	6	0	0.053963	5.850325	0.515925
94	6	0	-2.051074	5.363443	0.459150
95	6	0	-3.405404	4.987321	0.975955
96	7	0	-0.926575	5.127695	1.344054
97	6	0	-0.710889	7.140065	0.177779
98	8	0	-2.022507	6.799603	0.170225
99	8	0	-0.291138	8.232777	-0.076000
100	6	0	1.402706	6.125286	1.132485
101	1	0	1.902827	5.200541	1.426074
102	1	0	2.028180	6.663403	0.415709
103	1	0	1.290528	6.772475	2.006313
104	6	0	-3.566172	3.781907	1.666175
105	6	0	-4.517134	5.782713	0.703737
106	6	0	-5.784813	5.366080	1.107292
107	6	0	-4.831160	3.370504	2.068208
108	6	0	-5.944530	4.160688	1.784133
109	1	0	-4.386893	6.721381	0.176298
110	1	0	-6.647840	5.988378	0.891805
111	1	0	-2.694971	3.161043	1.861421
112	1	0	-4.949288	2.427857	2.593060
113	1	0	-6.933440	3.836740	2.093600
114	6	0	-1.078728	5.666029	2.695326
115	1	0	-1.914587	5.166059	3.186419
116	1	0	-0.170637	5.450147	3.261289
117	1	0	-1.262599	6.751518	2.714216

I-b

Center Number	Atomic Number	Atomic Type	Coordinates (Angstroms) X	Y	Z
1	6	0	-7.309851	0.221924	-0.613150
2	6	0	-8.008872	-1.012988	-0.798538
3	6	0	-5.166898	-1.001912	-0.531565

4	6	0	-5.885024	-2.239482	-0.762397
5	6	0	-3.039693	-2.237762	-0.455087
6	6	0	-8.036153	1.450257	-0.560547
7	6	0	-5.211177	1.450379	-0.333045
8	6	0	-5.943706	2.686736	-0.295656
9	6	0	-3.104952	2.683239	-0.095873
10	6	0	-5.901599	0.227861	-0.487626
11	6	0	-3.072101	0.229024	-0.193976
12	6	0	-3.785487	-1.005733	-0.366029
13	1	0	0.889802	7.376783	0.050009
14	6	0	-3.798606	1.457807	-0.219542
15	6	0	-0.986943	1.451712	0.009007
16	6	0	-1.684179	0.235956	0.006163
17	6	0	1.179857	0.271043	-0.150689
18	6	0	-3.759822	-3.457497	-0.769567
19	6	0	-0.925092	-3.437611	-0.549975
20	6	0	-1.642543	-4.661504	-0.850282
21	6	0	1.199013	-4.623273	-0.989867
22	6	0	-1.670237	-2.257480	-0.263398
23	6	0	1.209508	-2.216801	-0.428917
24	6	0	0.477783	-3.419311	-0.625506
25	6	0	3.287709	-3.364848	-1.072295
26	6	0	0.343458	6.437785	0.027207
27	6	0	3.274528	-0.909012	-0.691374
28	6	0	2.557778	-2.159614	-0.728278
29	6	0	5.383344	-2.097147	-1.154721
30	6	0	-3.845811	3.926764	-0.072894
31	6	0	-0.991887	3.933568	-0.014161
32	6	0	-1.743637	5.175178	0.039650
33	6	0	1.111461	5.212258	-0.091025
34	6	0	-1.701671	2.684512	-0.033533
35	6	0	1.127655	2.720903	-0.173619
36	6	0	0.453950	1.469119	-0.069092
37	6	0	3.232008	1.553080	-0.554685
38	6	0	0.390628	3.951090	-0.080229
39	6	0	3.229243	4.019809	-0.381782
40	6	0	2.520247	2.757682	-0.362176
41	6	0	5.341167	2.833115	-0.729787
42	6	0	2.540812	0.306517	-0.482694
43	6	0	5.350674	0.374180	-0.909222
44	6	0	4.645368	-0.873331	-0.914260
45	6	0	7.475871	-0.821934	-1.267298
46	6	0	4.636702	1.580313	-0.738781
47	6	0	7.452426	1.645084	-1.059368
48	6	0	6.753680	0.399409	-1.076991
49	6	0	-0.885676	-5.874004	-1.094734
50	1	0	-1.442377	-6.794170	-1.248504
51	6	0	-3.004918	-4.659414	-0.921562
52	1	0	-3.537554	-5.584396	-1.129724
53	6	0	-5.129286	-3.441548	-0.885031
54	1	0	-5.659394	-4.370302	-1.082498
55	6	0	-7.259575	-2.220229	-0.879437
56	1	0	-7.792683	-3.153022	-1.048474
57	6	0	-9.403189	-0.991430	-0.912161
58	1	0	-9.935322	-1.928014	-1.053288
59	6	0	-10.109359	0.213551	-0.848440
60	1	0	-11.191153	0.203910	-0.937907
61	6	0	-9.445948	1.414123	-0.678052
62	1	0	-9.999110	2.348063	-0.634405
63	6	0	-7.328150	2.659790	-0.400235
64	1	0	-7.883526	3.594255	-0.364665
65	6	0	-5.216250	3.906139	-0.158226
66	1	0	-5.770037	4.841922	-0.136618
67	6	0	-3.106258	5.153556	0.024570
68	1	0	-3.661911	6.087973	0.060294
69	6	0	-1.000418	6.420463	0.088601
70	1	0	-1.566552	7.345055	0.161855
71	6	0	0.455930	-5.856540	-1.161033
72	1	0	1.018760	-6.762108	-1.369718
73	6	0	2.552212	-4.585101	-1.170246

74	1	0	3.090267	-5.497651	-1.416734
75	6	0	4.651242	-3.317694	-1.242259
76	1	0	5.195398	-4.235082	-1.455199
77	6	0	6.752975	-2.047597	-1.312578
78	1	0	7.303274	-2.969177	-1.488547
79	6	0	8.865238	-0.769259	-1.421816
80	1	0	9.415281	-1.694785	-1.567480
81	6	0	9.544456	0.452866	-1.394410
82	1	0	10.623082	0.466921	-1.516352
83	6	0	8.858833	1.639928	-1.219009
84	1	0	9.391141	2.586812	-1.203234
85	6	0	6.721632	2.838794	-0.884905
86	1	0	7.255546	3.786309	-0.872089
87	6	0	4.591870	4.034245	-0.553865
88	1	0	5.122201	4.983822	-0.560855
89	6	0	2.467973	5.226608	-0.222062
90	1	0	2.999915	6.175407	-0.228565
91	6	0	0.581376	-1.046192	0.287106
92	6	0	-1.002165	-1.060015	0.385151
93	6	0	-1.200260	-1.191406	1.994398
94	6	0	0.957205	-1.236118	1.866576
95	6	0	2.383204	-1.007097	2.262812
96	7	0	-0.056182	-0.408533	2.475882
97	6	0	-0.752009	-2.620942	2.315617
98	8	0	0.599418	-2.604227	2.199758
99	8	0	-1.395616	-3.586324	2.605541
100	6	0	-2.540836	-0.861195	2.604769
101	1	0	-2.781613	0.198106	2.491963
102	1	0	-3.322272	-1.466121	2.141526
103	1	0	-2.527584	-1.117296	3.667564
104	6	0	2.863565	0.292553	2.442572
105	6	0	3.263917	-2.087617	2.346475
106	6	0	4.620338	-1.864271	2.564378
107	6	0	4.219370	0.512136	2.659563
108	6	0	5.102095	-0.565715	2.707137
109	1	0	2.886576	-3.095333	2.209194
110	1	0	5.303474	-2.706907	2.603838
111	1	0	2.176736	1.133103	2.362817
112	1	0	4.588480	1.527631	2.767498
113	1	0	6.163808	-0.391311	2.852780
114	6	0	0.019796	-0.300620	3.934443
115	1	0	0.961132	0.176616	4.207289
116	1	0	-0.799757	0.332117	4.279200
117	1	0	-0.038370	-1.273871	4.445602

I-c1

Center Number	Atomic Number	Atomic Type	Coordinates (Angstroms) X	Y	Z
1	6	0	8.671103	-0.180159	0.605944
2	6	0	9.423050	1.031939	0.790480
3	6	0	6.601975	1.138576	0.375035
4	6	0	7.363654	2.347418	0.559128
5	6	0	4.536876	2.454792	0.146091
6	6	0	9.340696	-1.452943	0.630387
7	6	0	6.520383	-1.319299	0.216556
8	6	0	7.200335	-2.589723	0.240832
9	6	0	4.373708	-2.455372	-0.171764
10	6	0	7.266878	-0.120293	0.398488
11	6	0	4.459549	-0.000554	-0.009999
12	6	0	5.208089	1.201201	0.168637
13	6	0	2.397388	1.317654	-0.234595
14	6	0	5.126409	-1.262395	0.009476
15	6	0	2.315616	-1.139008	-0.395286
16	6	0	3.063517	0.059591	-0.218900
17	6	0	0.255182	0.179025	-0.615334
18	6	0	5.296722	3.660902	0.328352
19	6	0	2.473388	3.773455	-0.078170
20	6	0	3.240471	4.982745	0.104166

21	6	0	0.401053	5.104025	-0.293384
22	6	0	3.146269	2.516859	-0.057675
23	6	0	0.337274	2.638098	-0.451579
24	6	0	1.087393	3.833706	-0.274398
25	6	0	-1.738347	3.964493	-0.649213
26	6	0	1.002044	1.379575	-0.438415
27	6	0	-1.804047	1.499647	-0.814576
28	6	0	-1.063189	2.696873	-0.640610
29	6	0	-3.876516	2.822359	-0.965105
30	6	0	5.051697	-3.722389	-0.149460
31	6	0	2.229134	-3.593676	-0.560184
32	6	0	2.913626	-4.864124	-0.538698
33	6	0	0.076655	-4.743908	-0.952606
34	6	0	2.983166	-2.398254	-0.377684
35	6	0	0.174815	-2.280035	-0.782023
36	6	0	0.920968	-1.081194	-0.602353
37	6	0	-1.884636	-0.962962	-0.998327
38	6	0	0.843294	-3.535686	-0.762847
39	6	0	-1.977364	-3.427302	-1.174842
40	6	0	-1.223756	-2.219910	-0.985835
41	6	0	-4.026733	-2.110052	-1.396486
42	6	0	-1.139189	0.238489	-0.811489
43	6	0	-3.955759	0.357162	-1.166598
44	6	0	-3.209199	1.558163	-0.979835
45	6	0	-6.030199	1.669235	-1.148552
46	6	0	-3.285515	-0.904689	-1.190252
47	6	0	-6.086852	-0.798861	-1.532513
48	6	0	-5.362675	0.411744	-1.279649
49	6	0	2.530092	6.245278	0.074851
50	1	0	3.108674	7.155685	0.209300
51	6	0	4.596093	4.908309	0.297885
52	1	0	5.168020	5.824221	0.434873
53	6	0	6.664898	3.585531	0.527147
54	1	0	7.232599	4.503576	0.664997
55	6	0	8.736900	2.267801	0.760218
56	1	0	9.305978	3.184004	0.899025
57	6	0	10.815224	0.940470	0.994023
58	1	0	11.386355	1.853459	1.133406
59	6	0	11.442595	-0.297842	1.014675
60	1	0	12.517280	-0.343760	1.172978
61	6	0	10.734967	-1.479349	0.838184
62	1	0	11.244332	-2.438206	0.857301
63	6	0	8.574926	-2.626945	0.444921
64	1	0	9.081807	-3.588835	0.462926
65	6	0	6.421145	-3.764161	0.052596
66	1	0	6.925880	-4.728009	0.068851
67	6	0	4.270278	-4.905639	-0.339495
68	1	0	4.779276	-5.867652	-0.324404
69	6	0	2.122291	-6.061954	-0.733121
70	1	0	2.638483	-7.018543	-0.718988
71	6	0	1.195237	6.304032	-0.111952
72	1	0	0.680467	7.260927	-0.131141
73	6	0	-0.959367	5.147964	-0.475462
74	1	0	-1.467992	6.109388	-0.488170
75	6	0	-3.124651	3.996886	-0.810682
76	1	0	-3.636219	4.956863	-0.806892
77	6	0	-5.302024	2.829682	-1.044207
78	1	0	-5.811970	3.790710	-0.998774
79	1	0	-1.850289	-5.587631	-1.298899
80	1	0	0.212320	-6.917162	-1.074905
81	6	0	-1.281172	-4.672585	-1.149606
82	6	0	0.788247	-6.007271	-0.928180
83	6	0	-7.523791	-0.689957	-1.754952
84	1	0	-8.044982	-1.563762	-2.139063
85	6	0	-5.433563	-2.005414	-1.602649
86	1	0	-5.999692	-2.912058	-1.807084
87	6	0	-3.357827	-3.343057	-1.378219
88	1	0	-3.927896	-4.255743	-1.538680
89	6	0	-8.191994	0.452465	-1.543765
90	6	0	-7.525625	1.658076	-0.935003

91	1	0	-9.256895	0.516939	-1.749879
92	1	0	-7.986966	2.579807	-1.308070
93	6	0	-7.822826	1.702727	0.656605
94	6	0	-7.534484	-0.360247	1.584929
95	6	0	-6.961985	-1.616225	2.009913
96	7	0	-6.920014	0.774331	1.359830
97	6	0	-9.147726	1.012611	0.904954
98	8	0	-8.853039	-0.283606	1.370362
99	8	0	-10.258814	1.377111	0.724489
100	6	0	-7.817334	3.113570	1.217995
101	1	0	-6.897032	3.633394	0.944153
102	1	0	-8.667519	3.650273	0.789991
103	1	0	-7.923674	3.111011	2.306588
104	6	0	-5.643100	-1.937034	1.645313
105	6	0	-7.766487	-2.545588	2.689538
106	6	0	-7.231186	-3.774951	3.037652
107	6	0	-5.128498	-3.182398	1.979175
108	6	0	-5.916774	-4.091680	2.682897
109	1	0	-8.792058	-2.292656	2.937887
110	1	0	-7.837864	-4.494509	3.576402
111	1	0	-5.043046	-1.246351	1.060576
112	1	0	-4.121059	-3.441665	1.669854
113	1	0	-5.509539	-5.062207	2.947920
114	6	0	-5.587515	1.143658	1.853737
115	1	0	-4.826027	1.034151	1.081850
116	1	0	-5.630591	2.185312	2.172466
117	1	0	-5.355416	0.510452	2.710464

I-c2

Center Number	Atomic Number	Atomic Type	Coordinates (Angstroms) X	Y	Z
1	6	0	-9.131837	0.161048	0.425488
2	6	0	-9.869966	-1.060284	0.501089
3	6	0	-7.045272	-1.118988	0.248147
4	6	0	-7.792231	-2.345929	0.321329
5	6	0	-4.957829	-2.400556	0.069162
6	6	0	-9.814828	1.416112	0.476729
7	6	0	-6.985227	1.344254	0.224154
8	6	0	-7.680195	2.605613	0.273402
9	6	0	-4.841853	2.531350	0.022861
10	6	0	-7.722951	0.127817	0.299143
11	6	0	-4.902819	0.063764	0.052892
12	6	0	-5.640189	-1.154373	0.121512
13	6	0	-2.820701	-1.216614	-0.120178
14	6	0	-5.586409	1.314554	0.098225
15	6	0	-2.759469	1.249910	-0.142703
16	6	0	-3.500234	0.031316	-0.075622
17	6	0	-0.677549	-0.031987	-0.308377
18	6	0	-5.710824	-3.629969	0.138584
19	6	0	-2.866766	-3.684248	-0.114993
20	6	0	-3.629569	-4.915383	-0.047674
21	6	0	-0.772477	-4.977974	-0.306420
22	6	0	-3.559216	-2.434054	-0.057640
23	6	0	-0.734760	-2.496711	-0.300771
24	6	0	-1.480774	-3.714759	-0.240358
25	6	0	1.362175	-3.785329	-0.493383
26	6	0	-1.414962	-1.249219	-0.247516
27	6	0	1.401828	-1.312940	-0.476342
28	6	0	0.667948	-2.526003	-0.423637
29	6	0	3.491119	-2.592984	-0.659747
30	6	0	-5.543367	3.794392	0.068636
31	6	0	-2.698465	3.721214	-0.182126
32	6	0	-3.409027	4.985790	-0.138129
33	6	0	-0.554889	4.922180	-0.393959
34	6	0	-3.450133	2.501534	-0.102972
35	6	0	-0.615982	2.437871	-0.345348
36	6	0	-1.362700	1.217915	-0.270116
37	6	0	1.471269	1.154672	-0.503611

38	6	0	-1.316399	3.690272	-0.306487
39	6	0	1.527137	3.636521	-0.561504
40	6	0	0.774161	2.405024	-0.470036
41	6	0	3.613633	2.354297	-0.728751
42	6	0	0.724171	-0.065513	-0.428701
43	6	0	3.558620	-0.132460	-0.642784
44	6	0	2.814754	-1.343548	-0.585300
45	6	0	5.633752	-1.417429	-0.750892
46	6	0	2.863683	1.119351	-0.619941
47	6	0	5.713161	1.072665	-0.887240
48	6	0	4.966293	-0.175838	-0.726881
49	6	0	-2.898472	-6.168062	-0.115124
50	1	0	-3.470144	-7.090779	-0.063973
51	6	0	-4.988954	-4.870776	0.075160
52	1	0	-5.555811	-5.797747	0.127204
53	6	0	-7.082484	-3.580928	0.262255
54	1	0	-7.649105	-4.507673	0.316767
55	6	0	-9.176008	-2.289607	0.446106
56	1	0	-9.743675	-3.215509	0.503094
57	6	0	-11.275458	-0.996002	0.627760
58	1	0	-11.839639	-1.922496	0.685950
59	6	0	-11.928074	0.226208	0.677415
60	1	0	-13.008868	0.253846	0.775023
61	6	0	-11.214841	1.420799	0.603219
62	1	0	-11.736643	2.372773	0.642753
63	6	0	-9.060015	2.614908	0.397622
64	1	0	-9.585749	3.566241	0.436180
65	6	0	-6.913448	3.808203	0.191307
66	1	0	-7.438756	4.759903	0.227608
67	6	0	-4.768199	5.001972	-0.016717
68	1	0	-5.294730	5.953249	0.017516
69	6	0	-2.626702	6.205694	-0.226848
70	1	0	-3.159476	7.152168	-0.192980
71	6	0	-1.557403	-6.197508	-0.237468
72	1	0	-1.026678	-7.144403	-0.287750
73	6	0	0.588033	-4.992842	-0.430444
74	1	0	1.113496	-5.943865	-0.482824
75	6	0	2.738978	-3.793740	-0.616407
76	1	0	3.265754	-4.743661	-0.673378
77	6	0	4.897290	-2.589263	-0.760264
78	1	0	5.411147	-3.545065	-0.834313
79	1	0	1.369022	5.804152	-0.586467
80	1	0	-0.714418	7.097537	-0.414057
81	6	0	0.803211	4.877608	-0.517575
82	6	0	-1.285996	6.175735	-0.347520
83	6	0	7.057679	1.055818	-1.093308
84	1	0	7.570002	1.994616	-1.289051
85	6	0	4.980807	2.306285	-0.876364
86	1	0	5.541064	3.232658	-0.981802
87	6	0	2.894451	3.589858	-0.691115
88	1	0	3.458027	4.516851	-0.769013
89	6	0	7.908386	-0.161827	-1.008698
90	6	0	7.145353	-1.503761	-0.773380
91	1	0	8.569511	-0.219342	-1.881123
92	1	0	7.425945	-2.243595	-1.530856
93	6	0	7.758912	-1.961271	0.604679
94	6	0	8.836630	-0.119460	0.256951
95	6	0	9.509427	1.182169	0.572311
96	7	0	7.956062	-0.663668	1.274162
97	6	0	9.200700	-2.331862	0.220366
98	8	0	9.843580	-1.153168	0.022669
99	8	0	9.699523	-3.409691	0.067422
100	6	0	7.066812	-3.042868	1.395188
101	1	0	6.060908	-2.735915	1.688861
102	1	0	7.006314	-3.952659	0.792639
103	1	0	7.647806	-3.289395	2.287694
104	6	0	8.778135	2.196442	1.199504
105	6	0	10.837865	1.405257	0.215087
106	6	0	11.431570	2.637505	0.481851
107	6	0	9.376177	3.422996	1.466047

108	6	0	10.704400	3.646276	1.105749
109	1	0	11.403245	0.612110	-0.261664
110	1	0	12.467275	2.805274	0.203594
111	1	0	7.743195	2.009995	1.475339
112	1	0	8.806059	4.204405	1.958767
113	1	0	11.170732	4.603992	1.314998
114	6	0	8.544700	-0.766006	2.608899
115	1	0	8.792170	0.234322	2.966845
116	1	0	7.804035	-1.197022	3.285105
117	1	0	9.456960	-1.382159	2.638708

G-Mu-a

Center Number	Atomic Number	Atomic Type	Coordinates (Angstroms) X	Y	Z
1	6	0	7.334789	1.244072	0.009431
2	6	0	7.598594	2.648185	-0.003127
3	6	0	4.918913	1.700572	-0.012684
4	6	0	5.193443	3.113132	-0.023713
5	6	0	2.500743	2.160853	-0.035461
6	6	0	8.418642	0.312807	0.026517
7	6	0	5.736041	-0.621144	0.017876
8	6	0	6.832672	-1.554319	0.036246
9	6	0	4.142000	-2.488811	0.027683
10	6	0	5.998442	0.775527	0.005654
11	6	0	3.324850	-0.161821	-0.004466
12	6	0	3.587381	1.237491	-0.014909
13	6	0	0.909551	0.304705	-0.020575
14	6	0	4.408108	-1.089116	0.016882
15	6	0	1.734441	-2.026126	0.005720
16	6	0	1.992834	-0.624763	0.000912
17	6	0	-0.674514	-1.569507	-0.014120
18	6	0	2.787102	3.573256	-0.046197
19	6	0	0.074388	2.645687	-0.047299
20	1	0	-0.528393	-7.761831	0.038633
21	6	0	-0.325420	-6.693134	0.031594
22	6	0	1.171187	1.711358	-0.031899
23	6	0	-1.506277	0.762890	-0.028906
24	6	0	-1.242743	2.179919	-0.027090
25	6	0	-3.932748	1.193212	-0.065917
26	6	0	-0.417635	-0.165200	-0.014160
27	6	0	-3.085488	-1.125959	-0.039645
28	6	0	-2.822200	0.275492	-0.039387
29	6	0	-5.513446	-0.678055	-0.081356
30	6	0	5.245603	-3.421988	0.046534
31	6	0	2.551027	-4.361725	0.036053
32	6	0	3.663627	-5.293371	0.054665
33	6	0	0.964241	-6.249261	0.040811
34	6	0	2.822055	-2.953647	0.027799
35	6	0	0.146381	-3.901303	0.011537
36	6	0	0.407991	-2.495804	0.008650
37	6	0	-2.259393	-3.450459	-0.015062
38	6	0	1.242925	-4.824956	0.032101
39	6	0	-1.442650	-5.790553	0.012307
40	6	0	-1.171953	-4.370728	0.006330
41	6	0	-3.851135	-5.337750	-0.020792
42	6	0	-2.001051	-2.047973	-0.016458
43	6	0	-4.668583	-3.001781	-0.049971
44	6	0	-4.411702	-1.603501	-0.055035
45	6	0	-7.090231	-2.553242	-0.090749
46	6	0	-3.585959	-3.922248	-0.026499
47	6	0	-6.258225	-4.884362	-0.058892
48	6	0	-6.001876	-3.478514	-0.066150
49	1	0	1.880392	-8.228187	0.067940
50	1	0	-2.943716	-7.306008	0.005850
51	6	0	1.681531	4.486895	-0.063419
52	1	0	1.923347	5.542799	-0.054559
53	6	0	4.088550	4.014526	-0.039636

54	1	0	4.291557	5.083109	-0.048286
55	6	0	6.508696	3.551831	-0.019127
56	1	0	6.714162	4.619843	-0.028222
57	6	0	8.937369	3.086836	0.001250
58	1	0	9.140408	4.154026	-0.008367
59	6	0	9.985040	2.173915	0.017477
60	1	0	11.009335	2.533651	0.020537
61	6	0	9.738099	0.806330	0.030131
62	1	0	10.563363	0.099976	0.042976
63	6	0	8.133609	-1.074235	0.039598
64	1	0	8.961921	-1.778995	0.052651
65	6	0	6.537549	-2.950347	0.049797
66	1	0	7.365835	-3.655186	0.062723
67	6	0	4.945225	-4.826697	0.059452
68	1	0	5.775470	-5.529478	0.072360
69	6	0	3.361227	-6.713138	0.066317
70	1	0	4.196553	-7.408071	0.079886
71	6	0	2.092416	-7.162428	0.059451
72	6	0	-2.743476	-6.236958	0.000235
73	6	0	-3.650650	2.599031	-0.061960
74	1	0	-4.499807	3.272193	-0.094365
75	6	0	-5.222336	0.718151	-0.087384
76	1	0	-6.050459	1.423179	-0.105906
77	6	0	-6.811708	-1.164918	-0.098348
78	1	0	-7.643079	-0.463928	-0.117969
79	6	0	-8.406788	-3.054711	-0.107018
80	1	0	-9.235915	-2.352975	-0.125880
81	6	0	-8.646597	-4.423538	-0.099219
82	1	0	-9.668866	-4.788764	-0.112043
83	6	0	-7.594120	-5.330916	-0.075642
84	1	0	-7.791136	-6.399239	-0.070048
85	6	0	-5.164231	-5.783142	-0.035938
86	1	0	-5.364707	-6.852116	-0.030776
87	6	0	-2.383371	3.094527	-0.021142
88	6	0	0.383161	4.075077	-0.076166
89	6	0	-2.085329	4.521326	-0.005774
90	6	0	-0.743602	4.994304	-0.088783
91	6	0	-0.788215	6.379004	-0.147622
92	6	0	-2.915854	5.630566	0.013249
93	6	0	-4.383888	5.748944	0.136073
94	7	0	-2.109120	6.746301	-0.079803
95	6	0	0.299879	7.396355	-0.282830
96	1	0	0.910828	7.465590	0.624823
97	1	0	0.966370	7.139480	-1.111971
98	1	0	-0.103009	8.390528	-0.487095
99	6	0	-2.577649	8.120138	-0.029539
100	6	0	-5.021085	5.368589	1.323416
101	6	0	-5.159176	6.234435	-0.922982
102	6	0	-6.542045	6.337867	-0.798406
103	6	0	-6.403694	5.462085	1.444133
104	6	0	-7.166559	5.949111	0.384039
105	1	0	-4.669230	6.518401	-1.850666
106	1	0	-7.132415	6.712960	-1.628672
107	1	0	-4.419191	4.985117	2.142470
108	1	0	-6.886251	5.159251	2.368231
109	1	0	-8.245294	6.024320	0.479566
110	1	0	-3.638643	8.121655	0.215429
111	1	0	-2.038401	8.674736	0.742105
112	1	0	-2.437529	8.618740	-0.993516

G-Mu-c

Center Number	Atomic Number	Atomic Type	Coordinates (Angstroms) X	Y	Z
1	6	0	8.902371	-0.091628	0.028363
2	6	0	9.628929	1.140980	0.024469
3	6	0	6.788612	1.161046	0.008743
4	6	0	7.524763	2.397451	0.003343
5	6	0	4.675333	2.414895	-0.010955

6	6	0	9.604367	-1.338710	0.040294
7	6	0	6.761585	-1.303159	0.024670
8	6	0	7.474970	-2.554495	0.035220
9	6	0	4.623042	-2.516437	0.021606
10	6	0	7.486295	-0.077437	0.019772
11	6	0	4.653492	-0.049949	0.008006
12	6	0	5.378719	1.177761	-0.001339
13	6	0	2.545096	1.203515	-0.009687
14	6	0	5.354701	-1.291833	0.014682
15	6	0	2.517267	-1.262811	0.007001
16	6	0	3.243876	-0.035966	-0.005966
17	6	0	0.410250	-0.008482	-0.009653
18	6	0	5.416597	3.652996	-0.018049
19	6	0	2.559873	3.671432	-0.031986
20	6	0	3.310397	4.911788	-0.039429
21	6	0	0.441560	4.938959	-0.054477
22	6	0	3.272234	2.430243	-0.021808
23	6	0	0.433779	2.457258	-0.030269
24	6	0	1.168445	3.684564	-0.040936
25	6	0	-1.686374	3.720199	-0.052847
26	6	0	1.134969	1.217804	-0.023621
27	6	0	-1.696607	1.245526	-0.026625
28	6	0	-0.972248	2.468494	-0.038698
29	6	0	-3.810396	2.502281	-0.042445
30	6	0	5.341796	-3.768941	0.030632
31	6	0	2.484197	-3.732718	0.018436
32	6	0	3.211776	-4.987355	0.027398
33	6	0	0.344720	-4.961117	0.015702
34	6	0	3.223330	-2.504566	0.011070
35	6	0	0.379872	-2.477158	0.004020
36	6	0	1.111315	-1.249274	-0.007263
37	6	0	-1.728620	-1.221383	-0.011445
38	6	0	1.094722	-3.719569	0.010459
39	6	0	-1.759710	-3.702925	0.001083
40	6	0	-1.020422	-2.461687	-0.004997
41	6	0	-3.863715	-2.446806	-0.013285
42	6	0	-0.998239	0.006249	-0.020990
43	6	0	-3.842137	0.036557	-0.023360
44	6	0	-3.110737	1.257325	-0.029815
45	6	0	-5.947872	1.307733	-0.013628
46	6	0	-3.133602	-1.204194	-0.018303
47	6	0	-5.978380	-1.198901	-0.028592
48	6	0	-5.257217	0.057888	-0.021661
49	6	0	2.561151	6.155204	-0.053993
50	1	0	3.123939	7.084898	-0.058758
51	6	0	4.675817	4.884140	-0.032045
52	1	0	5.233063	5.818560	-0.036450
53	6	0	6.794029	3.622277	-0.010090
54	1	0	7.350339	4.557002	-0.013880
55	6	0	8.913472	2.360635	0.011723
56	1	0	9.470797	3.294540	0.008520
57	6	0	11.039374	1.096201	0.033090
58	1	0	11.594140	2.030076	0.030250
59	6	0	11.709331	-0.119106	0.044990
60	1	0	12.795176	-0.131132	0.051483
61	6	0	11.011970	-1.322980	0.048482
62	1	0	11.548126	-2.267637	0.057701
63	6	0	8.861575	-2.545610	0.043152
64	1	0	9.400319	-3.490321	0.052094
65	6	0	6.718303	-3.765365	0.037828
66	1	0	7.255949	-4.710921	0.046261
67	6	0	4.576987	-4.985766	0.033406
68	1	0	5.116082	-5.930752	0.041339
69	6	0	2.440174	-6.217038	0.030621
70	1	0	2.985873	-7.156834	0.038116
71	6	0	1.214243	6.168278	-0.061081
72	1	0	0.669329	7.108448	-0.072287
73	6	0	-0.924501	4.937138	-0.061191
74	1	0	-1.464183	5.881624	-0.072886
75	6	0	-3.066983	3.711771	-0.057681

76	1	0	-3.607292	4.655851	-0.069519
77	6	0	-5.212146	2.493659	-0.039064
78	1	0	-5.723945	3.449082	-0.072534
79	1	0	-1.577915	-5.868196	0.013765
80	1	0	0.530463	-7.134497	0.028034
81	6	0	-1.020113	-4.934280	0.010744
82	6	0	1.093528	-6.205047	0.025439
83	6	0	-7.372622	-1.210056	-0.046731
84	1	0	-7.902201	-2.159423	-0.040619
85	6	0	-5.248312	-2.413102	-0.020326
86	1	0	-5.802592	-3.349172	-0.021425
87	6	0	-3.136039	-3.672124	-0.004092
88	1	0	-3.693773	-4.605926	-0.000902
89	6	0	-8.079251	0.002036	-0.050028
90	6	0	-7.393326	1.265658	0.006898
91	6	0	-8.364345	2.251133	0.087522
92	6	0	-9.457523	0.264356	-0.019423
93	6	0	-10.567717	-0.690932	-0.016359
94	7	0	-9.594965	1.624362	0.061302
95	6	0	-8.241453	3.735945	0.213892
96	1	0	-7.510305	3.988946	0.987416
97	1	0	-7.911923	4.204985	-0.720843
98	1	0	-9.187675	4.197722	0.503564
99	6	0	-11.622195	-0.593803	0.904285
100	6	0	-10.563782	-1.761955	-0.922064
101	6	0	-11.585144	-2.705343	-0.907073
102	6	0	-12.648848	-1.530828	0.907554
103	6	0	-12.633339	-2.590694	0.003273
104	1	0	-9.761394	-1.832925	-1.650700
105	1	0	-11.568823	-3.525958	-1.617537
106	1	0	-11.617417	0.206845	1.639050
107	1	0	-13.455346	-1.442226	1.628805
108	1	0	-13.432893	-3.324517	0.009993
109	6	0	-10.859361	2.340462	-0.011658
110	1	0	-11.635679	1.653309	-0.344333
111	1	0	-11.137892	2.751047	0.963631
112	1	0	-10.777156	3.155115	-0.733506

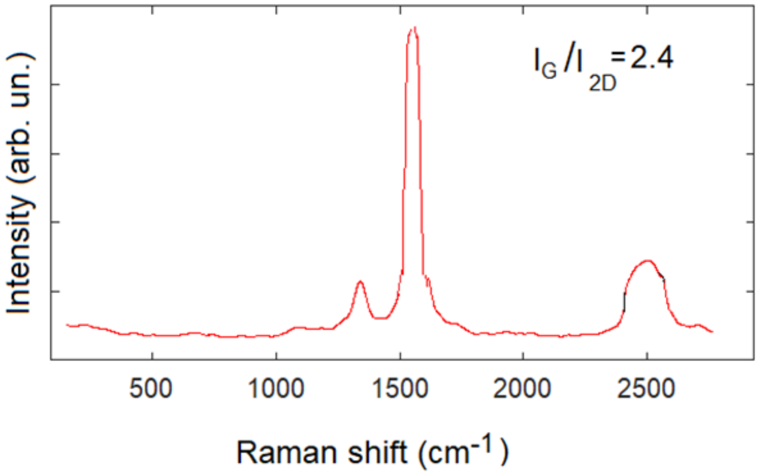

Figure S1. Raman spectrum of pristine graphite

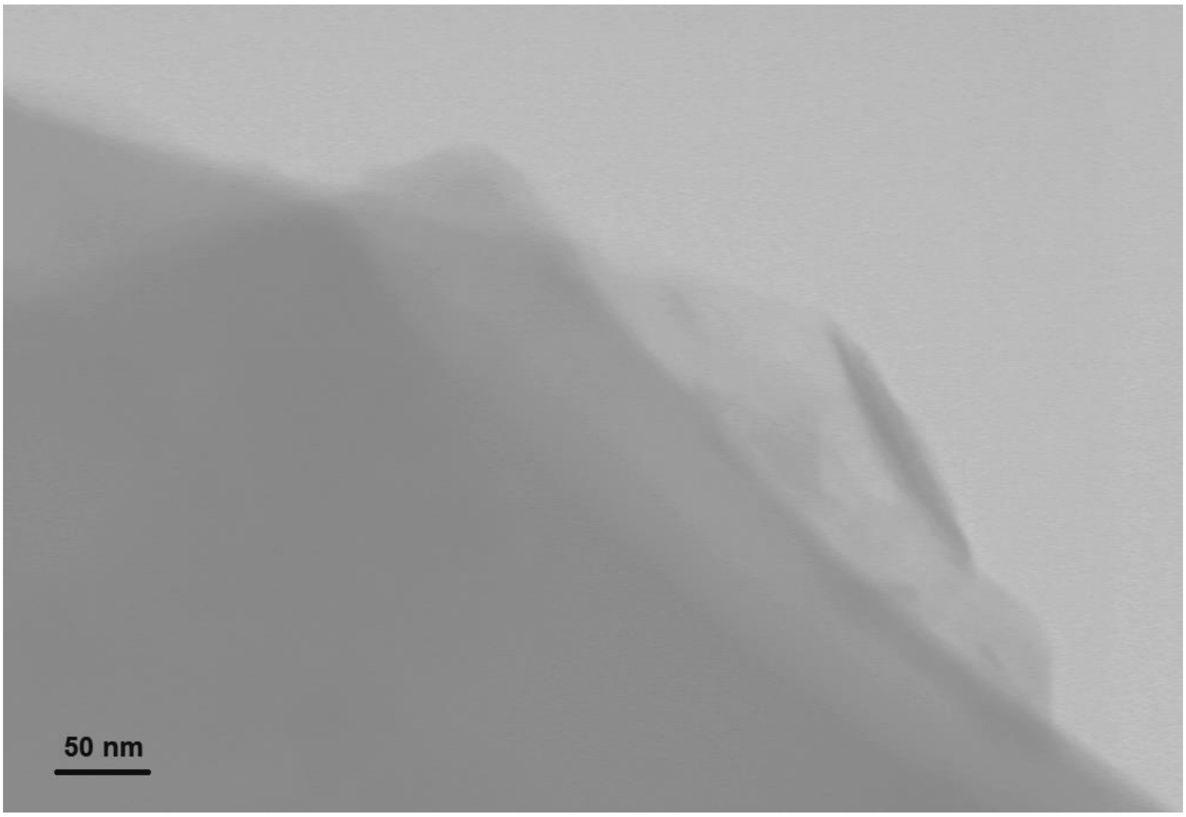

Figure S2. STEM image of G-MuSH
